# Lysophospholipid signaling coordinates outer membrane homeostasis in *Escherichia coli*

**DOI:** 10.1128/mbio.00567-26

**Published:** 2026-04-14

**Authors:** Gerald R. Enverso, M. Stephen Trent

**Affiliations:** 1 Department of Microbiology, College of Arts and Sciences, University of Georgia 138572, Athens, Georgia, USA; 2 Department of Infectious Diseases, College of Veterinary Medicine, University of Georgia 551782, Athens, Georgia, USA; The University of Texas Health Science Center at Houston John P. and Katherine G. McGovern Medical School, Houston, Texas, USA

**Keywords:** LplT, Aas, outer membrane, PldA, lysophospholipid, lipopolysaccharide, LpxC

## Abstract

The Gram-negative outer membrane (OM) is an asymmetric bilayer that protects cells from environmental stress and antibiotics. This asymmetry, with lipopolysaccharide (LPS) in the outer leaflet and glycerophospholipids (GPLs) in the inner leaflet, requires coordinated synthesis of both lipid classes. The committed step of LPS biosynthesis is catalyzed by LpxC, a prime antibiotic target. Here, we show that lysophospholipids (LPLs), considered byproducts of membrane turnover, act as signaling molecules restoring OM homeostasis when LPS synthesis is limited. In the presence of the LpxC inhibitor PF-5081090 (PF), loss of the LPL recycling system increased growth, suppressed envelope stress responses, improved OM asymmetry, and lowered GPL levels to maintain GPL-to-LPS balance. This recycling system includes the transporter LplT, which moves LPLs across the inner membrane, and the acyltransferase/acyl-ACP synthetase (Aas), which acylates them to regenerate GPLs. These protective effects required the OM phospholipase PldA that degrades mislocalized GPLs into LPLs and free fatty acids. Although previous work showed that PldA-generated fatty acids stabilize LpxC and promote LPS synthesis, our findings reveal a complementary role for LPLs in signaling reduced GPL synthesis when LPS is limiting. Genetic and chemical manipulation of fatty-acid flux altered PF resistance, confirming that decreased GPLs drives protection. The two PldA-derived signals, fatty acids that promote LPS synthesis and LPLs that suppress GPL synthesis, likely operate under different metabolic conditions to interpret membrane stress and restore OM balance. This lipid-feedback mechanism establishes the first signaling function for bacterial LPLs and reveals a new layer of regulation in envelope homeostasis.

**IMPORTANCE:**

The multilayered cell envelope of Gram-negative bacteria provides natural resistance to antibiotics. Understanding cell envelope synthesis and regulation is crucial for the identification of new antimicrobial targets and improved drug design. LpxC inhibitors, a new and promising class of antibiotics, impede function of the committed enzyme in lipopolysaccharide synthesis. Here, we characterize a new mechanism of resistance to the LpxC inhibitor PF-5081090, where the accumulation of lysophospholipids signals a reduction in cellular glycerophospholipid levels to repair outer membrane balance. This work proposes a new pathway to restore outer membrane asymmetry, which is a critical aspect of cell envelope integrity, and describes a role for lysophospholipids in bacterial cell signaling when lipopolysaccharide synthesis is disrupted.

## INTRODUCTION

**Fig 1:**
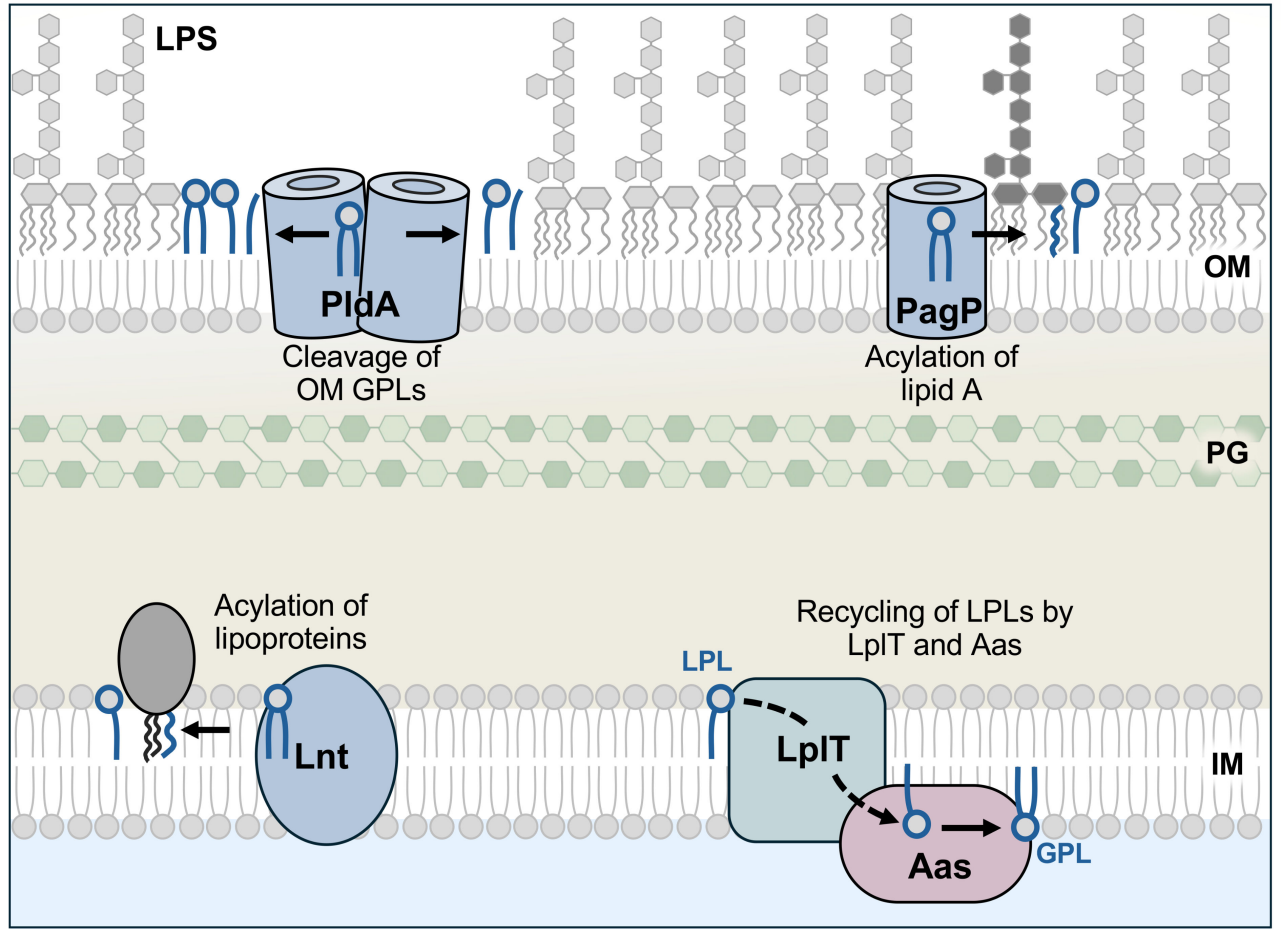
LPL recycling in *E. coli*. LPLs are generated through multiple processes in the cell envelope. During lipoprotein maturation at the IM, Lnt transfers an acyl chain from a GPL to a lipoprotein, which produces an LPL byproduct. When OM asymmetry is disrupted, the phospholipase PldA degrades mislocalized GPLs in the OM outer leaflet into LPLs and free fatty acids. The OM enzyme PagP transfers an acyl chain from surface GPLs to the lipid A anchor of LPS, also generating LPLs. During the process of LPL recycling, LPLs are transported from the periplasmic to the cytoplasmic leaflet of the IM by LplT, where Aas uses bound acyl-ACP to acylate LPLs to regenerate GPLs. IM, inner membrane; PG, peptidoglycan; OM, outer membrane.

Multidrug-resistant bacterial infections pose a serious and growing threat to global public health (1). Of particular concern are Gram-negative pathogens, as their unique cell envelope provides an effective permeability barrier against many antibiotics (2). The cell envelope of these organisms contains an inner and outer membrane separated by an aqueous periplasmic space that houses the peptidoglycan cell wall, which is critical for cell shape. The inner membrane (IM) is a symmetrical bilayer composed of glycerophospholipids (GPLs), whereas the outer membrane (OM) is asymmetrically organized with GPLs in the inner leaflet and mainly lipopolysaccharide (LPS) in the outer leaflet (3, 4) (Fig. 1). This lipid asymmetry is essential for OM barrier function and overall envelope stability (5).

**Fig 2:**
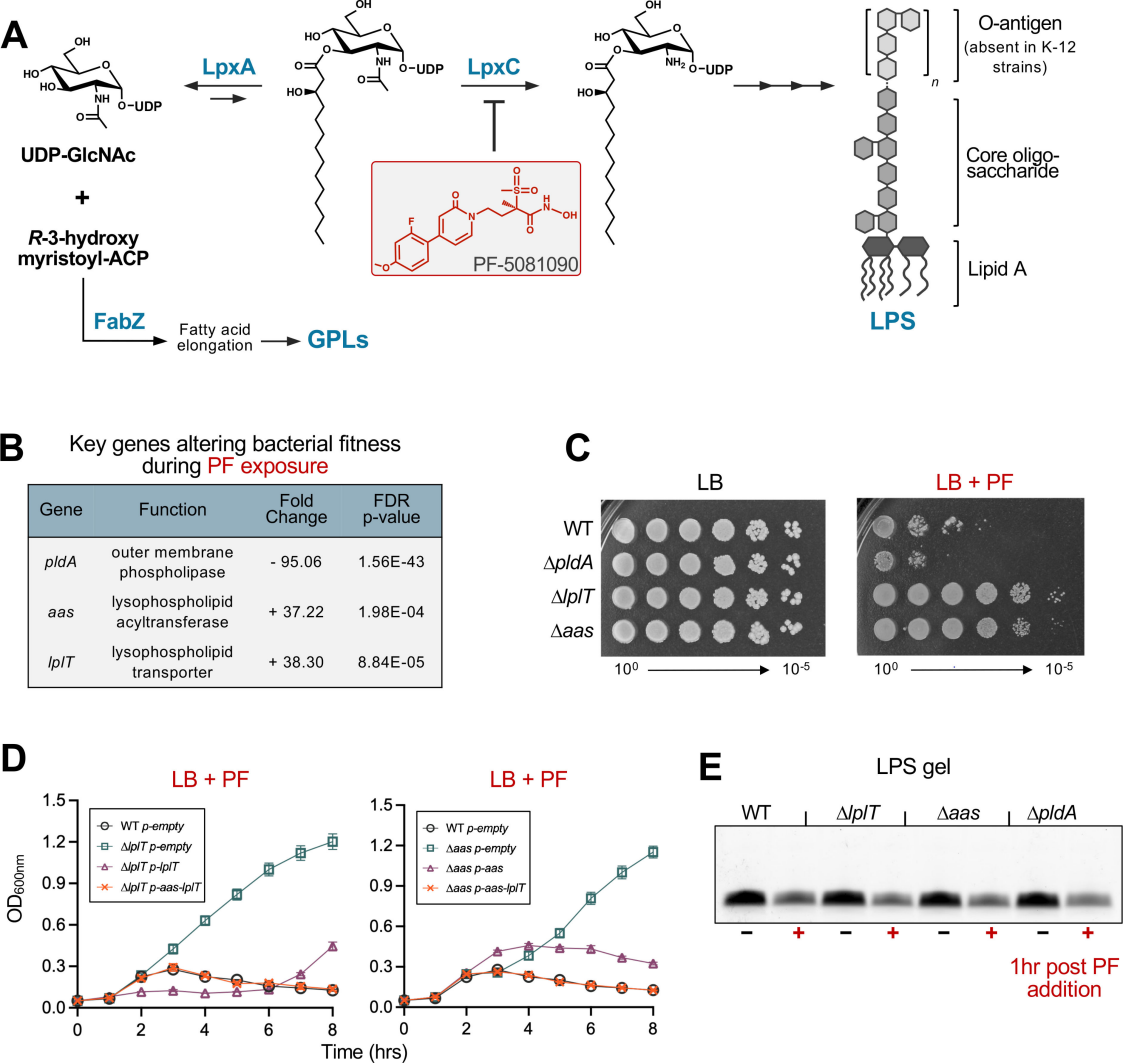
Disruption of LPL recycling confers resistance to LpxC inhibition. (**A**) Schematic featuring the early steps of LPS synthesis and competing fatty acid utilization. LpxA catalyzes the reversible acylation of UDP-GlcNAc using *R*-3-hydroxymyristoyl-ACP as the acyl donor, initiating lipid A synthesis. The deacetylase LpxC catalyzes the second and committed step of the pathway. In parallel, *R*-3-hydroxymyristoyl-ACP can enter the type II fatty acid synthesis cycle through FabZ, promoting further acyl-chain elongation and directing intermediates toward GPL production. Thus, LpxA and FabZ compete for a shared acyl-ACP pool. Subsequent enzymes complete assembly of lipid A, core oligosaccharide, and O-antigen polysaccharide to generate mature LPS. LPS synthesis is inhibited by PF-5081090, which binds the active site of LpxC and blocks enzyme activity. (**B**) Key genes altering fitness of a WT Tn-library in the presence of 20 ng/mL PF. A negative fold change indicates disruptions that are detrimental to fitness, while a positive fold change suggests disruptions that benefit fitness. FDR-corrected *P*-values are less than 0.05. (**C**) Efficiency of plating assay on LB and LB + 25 ng/mL PF plates. (**D**) Growth curves of WT and LPL-recycling mutants in LB with 20 ng/mL PF. Cultures were back-diluted to a starting OD_600_ of 0.05, and 100 µg/mL ampicillin was included for plasmid maintenance. For complementation, *p-aas* and *p-aas-lplT* were induced with 100 µM IPTG, whereas *p-lplT* required only basal (uninduced) expression. Error bars represent standard deviations (SD) and are not visible if smaller than the graphical symbol. (**E**) LPS levels following PF exposure. Cultures were grown to an OD_600_ of 0.4 and exposed to 40 ng/mL PF for 1 h, and LPS abundance was assessed by SDS-PAGE. Data are representative of a minimum of three biological replicates.

LPS can be divided into the following three parts: lipid A, core oligosaccharide, and O-antigen polysaccharide (Fig. 2A). In *Escherichia coli*, the lipid A domain anchors LPS within the membrane and consists of a glucosamine disaccharide that is *bis*-phosphorylated and hexa-acylated. The phosphate groups coordinate divalent cations, creating cross-bridges that stabilize the OM (6). Addition of the core oligosaccharide and O-antigen further strengthens lateral interactions between LPS molecules and enhances protection from various environmental stressors (7).

Although the core-oligosaccharide and O-antigen domains of LPS are dispensable, synthesis of the lipid A anchor is essential in most organisms. The first step of synthesis is reversible, and the second step, catalyzed by the deacetylase LpxC (Fig. 2A), serves as the committed step (8). Following the reaction of LpxC, seven additional steps are required to produce Kdo_2_-lipid A—the most basic structure of LPS required for functionality (4, 9). LpxC activity drives the consumption of *R*-3-hydroxymyristoyl-ACP, a fatty-acid intermediate that is also required by the enzyme FabZ for fatty acid elongation and downstream GPL synthesis (Fig. 2A). Because both pathways compete for this shared resource, changes in LpxC activity directly influence the balance of LPS and GPL production. Perturbations in this balance alter OM composition, thereby impacting barrier function (10).

In *E. coli*, LpxC levels are modulated by proteolysis through the LapB-FtsH complex, which itself is regulated by the essential membrane protein YejM. During logarithmic growth, when LPS biosynthesis is required, the accessory protein YejM binds to LapB (YciM) and inhibits the recruitment of LpxC to the FtsH protease for degradation. When LPS accumulates in the outer leaflet of the IM and binds to YejM, LapB is released to stimulate LpxC degradation (11–15). LapB has also been shown to directly bind to LpxC, inhibiting enzymatic function (16). This regulatory network maintains the proper balance of LPS and GPLs for OM integrity.

The conserved structure and essential function of LpxC make it an attractive target for antimicrobial development (17). Inhibition of LpxC not only blocks the production of an essential cell envelope component but also destabilizes the OM, rendering the cells hypersensitive to a wide range of antibiotics and to components of the mammalian immune system (18). Several generations of LpxC inhibitors, such as CHIR-090 and PF-5081090 (PF) (Fig. 2A), show potent, broad-spectrum activity against species such as *Pseudomonas aeruginosa* and *E. coli* (19). Investigating how resistance is acquired to LpxC inhibitors has advanced our understanding of cell envelope homeostasis. Mutations that increase drug efflux are critical for resistance to PF in *P. aeruginosa* (19). In *E. coli*, suppressor mutations that confer resistance to CHIR-090 reduce the activity of FabZ, thereby slowing GPL synthesis (Fig. 2A) and restoring the GPL-to-LPS ratio in the OM (20).

To uncover additional pathways that alter fitness when LPS synthesis is decreased, we performed a genome-wide transposon sequencing (Tn-seq) screen in *E. coli* K-12 strain W3110 in the absence and presence of the antibiotic PF. Consistent with prior work, insertions that disrupted drug efflux systems or reduced GPL synthesis affected bacterial fitness. Surprisingly, disruption of the *aas-lplT* operon, which encodes the lysophospholipid (LPL) recycling system, markedly increased fitness during LpxC inhibition. The enrichment of these mutants suggested that LPL metabolism influences the cellular response to reduced LPS synthesis. This finding raised the possibility that the accumulation or altered processing of LPLs may signal adjustments in lipid biosynthesis that help restore membrane balance during LpxC inhibition.

GPLs are the major structural lipids of bacterial membranes, whereas LPLs are mono-acylated byproducts generated during envelope remodeling. LPLs can arise through several enzymatic reactions across the cell envelope. At the IM, LPLs are generated during lipoprotein maturation, where the enzyme Lnt transfers an acyl chain from the *sn*-1 position of a GPL donor to a lipoprotein (21) (Fig. 1). In the OM, the phospholipase PldA and palmitoyltransferase PagP utilize mislocalized GPLs that have flipped to the outer leaflet as substrate and release LPLs as byproducts. While PldA cleaves GPLs into LPLs and fatty acids, PagP functions as an acyltransferase that transfers a palmitate from a GPL to lipid A to produce hepta-acylated LPS and an LPL (22–24) (Fig. 1). Like Lnt, both enzymes exhibit *sn*-1 position preference and generate a 2-acyl LPL.

Because the accumulation of LPLs perturbs membrane curvature and integrity (25), many bacteria possess a dedicated LPL recycling system comprised of an LPL transporter (LplT) and acyltransferase/acyl-ACP synthetase (Aas) (26) (Fig. 1). *In vitro*, LplT transports lyso-forms of the three major GPLs in *E. coli*—phosphatidylethanolamine (PE), phosphatidylglycerol (PG), and cardiolipin (CL)—from the outer to the inner surface of spheroplasts (27). An “alternating-access” mechanism of LplT is proposed as its 12 transmembrane domains cluster into two lobes that form an open, V-shaped groove on the periplasmic side of the protein when docked with lyso-PE (28). This work implicates LplT in the transport of LPLs from the periplasmic to the cytoplasmic leaflet of the IM. After transport across the IM, LPLs are regenerated into GPLs via Aas, which has both acyl-ACP synthetase and acyltransferase activity encoded by separate domains (29). This enzyme contains a high-affinity binding site for ACP and ligates free fatty acids to form bound acyl-ACP. Acyl-ACPs generated by *de novo* fatty acid biosynthesis can also be utilized as substrates *in vitro*, but to a lesser extent (30, 31). This bound acyl-ACP is used for the transfer of an acyl chain to LPLs in the IM. LplT and Aas likely function as a complex as LplT has been shown to interact directly with the acyltransferase domain of Aas *in vitro* (32), allowing direct transfer of LPLs from LplT to Aas to promote efficient recycling.

While previous work has emphasized the importance of LPL recycling in protecting membrane integrity, our findings reveal that LPLs themselves function as signaling molecules that coordinate membrane biogenesis. Here, we show that loss of the LplT-Aas recycling system unexpectedly increased growth during LpxC inhibition when LPS is limiting. This resistance depended on the OM phospholipase PldA, which produces LPLs. When LPL recycling is blocked, PldA drives LPL accumulation and triggers a feedback response that slows GPL biosynthesis, restoring the proper balance of LPS and GPLs in the OM. Manipulating fatty-acid flux genetically or by chemical means confirmed that this decrease in GPL production underlies the protective phenotype. Together, these results uncover a lipid-based feedback mechanism that allows bacteria to sense and correct disruptions in OM composition, establishing a new signaling role for LPLs in bacterial envelope homeostasis.

## RESULTS

### Disruption of LPL recycling confers resistance to LpxC inhibition

The first step of LPS synthesis is the reversible acylation of UDP-*N*-acetylglucosamine (UDP-GlcNAc) by LpxA, whereas the second step, catalyzed by the deacetylase LpxC, is the committed and rate-limiting step of the pathway (Fig. 2A). Given the essential nature of LPS for OM assembly, targeting LpxC has become a focal point for antimicrobial design. The inhibitor PF-5081090 (PF) is a sulfone hydroxamate, where the sulfone group interacts with the active site of LpxC, and the hydroxamate chelates the catalytic zinc ion (17, 33). Treatment with PF reduces LPS levels (34) and inhibits the growth of *E. coli* (19).

To identify genes important for fitness when LPS synthesis is reduced, we performed Tn-seq in the absence and presence of PF using a saturated *E. coli* Tn library in the K-12 strain W3110 (Data Set S1). Transposon insertions that would result in the truncation of the LPS core oligosaccharide (*rfa/waa* genes), lowered cellular levels of LPS (*yejM*), or disrupted drug efflux (*acrAB*, *tolC*) all negatively impacted fitness. Conversely, an enrichment of insertions throughout *fabF, fadR,* and the regions encoding the C-terminus of FabA and FabG (Data Set S1) suggested that slowing fatty acid and downstream GPL synthesis increases fitness when LPS production is limited. These data agree with previous work (19, 20) demonstrating that survival during LpxC inhibition requires balanced synthesis of LPS and GPLs.

During growth in PF, we observed ~95-fold fewer Tn insertions in *pldA* (Fig. 2B). PldA is an OM phospholipase that degrades mislocalized GPLs at the cell surface into LPLs and free fatty acids (22, 23). PldA function is likely required to respond to disrupted OM asymmetry during LpxC inhibition. Interestingly, an enrichment in Tn insertions throughout *aas* (+37-fold) and *lplT* (+38-fold) occurred in the presence of PF (Fig. 2B). LplT transports LPLs from the periplasmic to the cytoplasmic leaflet of the IM, where Aas acylates LPLs to regenerate GPLs (Fig. 1). These results suggest that PldA is crucial for fitness, while the function of LplT and Aas hinders growth during LpxC inhibition. We hypothesized that LPLs produced by PldA accumulate when LPL recycling is blocked and that this accumulation somehow promotes PF resistance.

To validate the Tn-seq screen, we constructed single-gene deletion mutants in the W3110 (WT) background by generalized phage transduction using the Keio collection (35). In rich media, ∆*lplT* or ∆*aas* exhibited no growth defect (Fig. S1A), and efficiency of plating assays indicated that both mutants were resistant to PF compared to WT (Fig. 2C). Deletion of the entire *aas-lplT* operon did not result in additive PF resistance compared to single mutants (Fig. S1B). Furthermore, neither mutant displayed increased resistance to other antibiotics or detergents (Fig. S1C), demonstrating that resistance is specific to PF and not due to a general decrease in cell permeability. PF sensitivity is largely restored in ∆*lplT* and ∆*aas* with expression of *lplT* or *aas* from an IPTG-inducible promoter, respectively. Expression of the entire *aas-lplT* operon fully restores growth to WT levels (Fig. 2D), likely reflecting either polar effects associated with single-gene deletions or the need for coordinated expression of LplT and Aas to maintain proper functional stoichiometry. However, expression of previously characterized, inactive variants of LplT (LplT_D30A_) (36) or Aas (Aas_H36A_) (37) failed to restore PF sensitivity in the corresponding mutant strains (Fig. S1D). These data suggest that loss of LplT and Aas activity, and not secondary effects of gene deletion, is implicated in PF resistance.

Because PF targets LpxC, we first determined whether resistance of LPL-recycling mutants reflects altered LpxC abundance or stability. LpxC levels were quantified by immunoblotting following growth in the absence or presence of PF. In WT cells, PF treatment caused a marked increase in LpxC abundance relative to DMSO-treated controls (Fig. S2A). This response is expected, as reduced LPS synthesis stabilizes LpxC through YejM-dependent modulation of the LapB-FtsH protease complex. Importantly, LPL-recycling mutants exhibited a comparable PF-induced increase in LpxC abundance, indicating that this homeostatic response remains intact in LPL-recycling backgrounds.

To test whether LPL-recycling mutants alter LpxC turnover, chloramphenicol was added after 2 h of PF exposure, and LpxC decay was monitored over time. LpxC stability was indistinguishable between WT, Δ*lplT,* and Δ*aas* (Fig. S2B). Thus, PF resistance of LPL-recycling mutants cannot be attributed to increased LpxC abundance or altered LpxC stability. We next examined whether resistance instead reflects restoration of LPS production downstream of LpxC inhibition. WT and LPL-recycling mutants displayed comparable LPS levels during growth in LB and exhibited a similar reduction in LPS following PF treatment for 1 h. Likewise, PF stress induced a decrease in LPS with loss of PldA (Fig. 2E). Together, these results indicate that resistance does not arise from increased LpxC levels or preservation of bulk LPS following PF exposure.

### Elimination of LPL recycling suppresses a membrane stress response when LPS is limited

To better understand how loss of LPL recycling confers resistance to LpxC inhibition, we performed RNA sequencing of WT and ∆*lplT* grown with or without PF (Data Set S2). During growth in LB, the only notable difference between the transcriptomes of WT and ∆*lplT* was the loss of *lplT* transcripts. Upon PF treatment, both WT and ∆*lplT* showed widespread transcriptional changes, many of which were shared and considered to be general cellular responses to LpxC inhibition.

**Fig 3:**
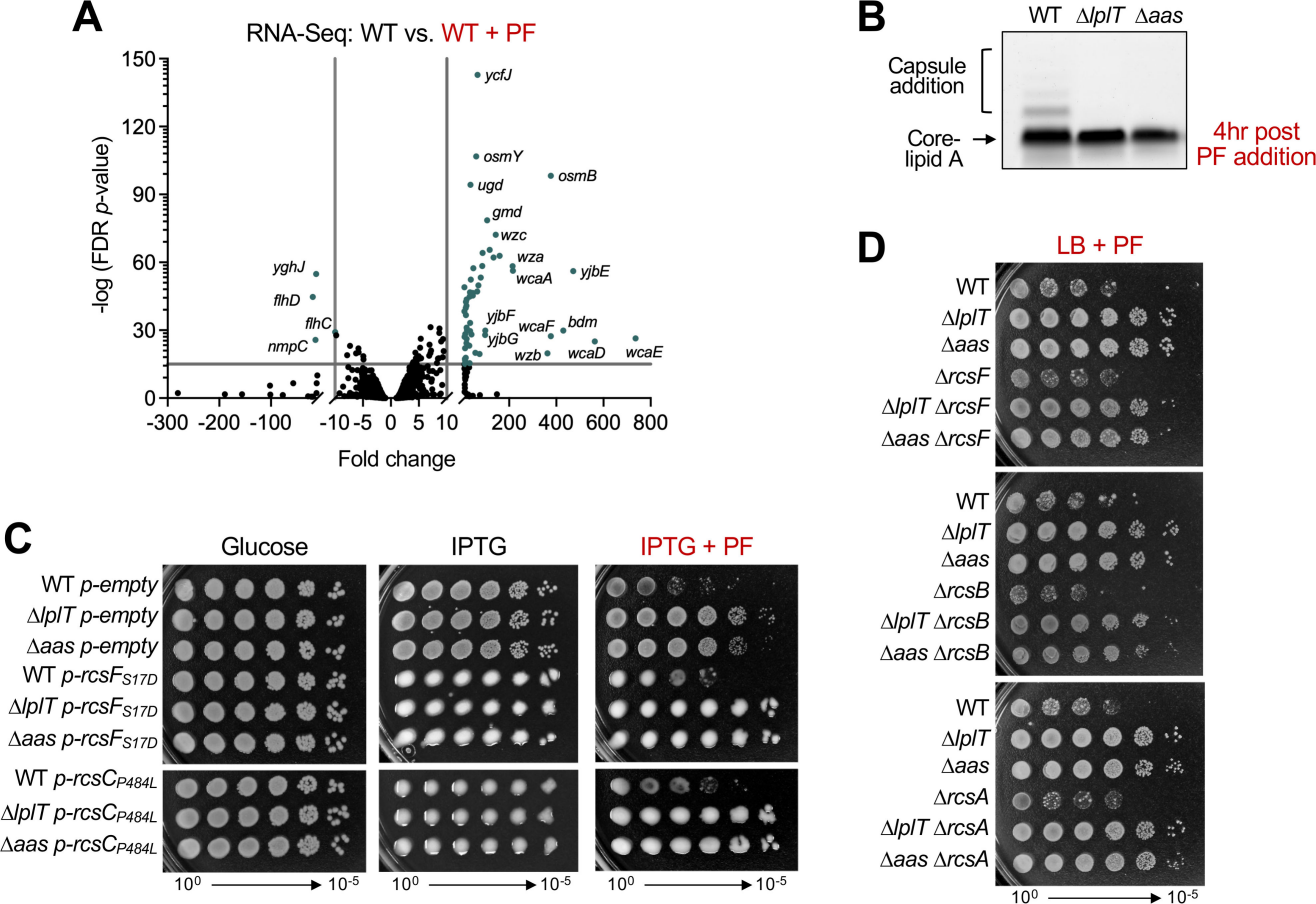
LPL-recycling mutants suppress activation of the Rcs envelope stress response during LpxC inhibition. (**A**) Volcano plot of RNA sequencing data comparing WT grown in LB versus LB with PF. PF was added to cultures at an OD_600_ of 0.2, and cultures were harvested at an OD_600_ of 0.5. Hits with a fold change ≥10 and a −log (FDR *P*-value) >15 are colored in teal. (**B**) LPS profiles of WT and LPL-recycling mutants following PF treatment. Cultures were grown to an OD_600_ of 0.4 and exposed to 40 ng/mL PF for 4 h, and changes in LPS were assessed by SDS-PAGE. Capsule addition to LPS is used as a measurement of Rcs induction and is indicated by a bracket. (**C**) Efficiency of plating assays of WT, ∆*lplT*, and ∆*aas* expressing hyperactive alleles of Rcs proteins. All plates contained 50 µg/mL ampicillin for plasmid maintenance. Where indicated, 0.2% glucose, 50 µM IPTG, or 25 ng/mL PF was included. (**D**) Efficiency of plating assays of ∆*rcsF,* ∆*rcsB,* or ∆*rcsA* mutants in WT, ∆*lplT*, and ∆*aas* backgrounds on LB + 25 ng/mL plates. Data are representative of a minimum of three biological replicates.

One clear distinction between the strains was activation of the regulator of capsule synthesis (Rcs) regulon in WT but not in ∆*lplT*. The Rcs signal transduction pathway responds to envelope damage, particularly defects in peptidoglycan or LPS structure, to increase capsule synthesis and biofilm formation while decreasing flagellar motility (38). In PF-treated WT cells, genes involved in extracellular polysaccharide biosynthesis (*yjbEFGH*, *wcaABCDEF*, *wzb*, etc.), osmotic stress response (*osmB*/*Y*), and the Rcs accessory activator *rcsA* were strongly upregulated. RcsA interacts with the Rcs response regulator to facilitate binding to promoter regions (39). Meanwhile, a clear downregulation of genes encoding the flagellar master regulator (*flhDC*) was detected (Fig. 3A; Data Set S2). In contrast, PF treatment failed to induce Rcs-regulated changes in ∆*lplT* (Fig. S3), indicating that loss of LPL recycling suppresses the envelope stress normally caused by LpxC inhibition.

Activation of the Rcs response induces the synthesis of colanic acid, or capsule, that can also be attached to lipid A-core of *E. coli* K-12 strains via the O-antigen ligase WaaL (39, 40). To confirm RNA sequencing results, we monitored capsule addition to LPS following PF exposure. Proteinase K-treated whole cell lysates of WT and LPL-recycling mutants were separated by SDS-PAGE, and LPS species were stained with Pro-Q Emerald 300 carbohydrate dye. After 4 h of antibiotic treatment, WT cells displayed capsule addition to LPS, consistent with Rcs activation, while *lplT* and *aas* mutants did not (Fig. 3B). To determine if hyperactivation of the Rcs response contributes directly to PF sensitivity, we altered key components of the Rcs system. The OM lipoprotein RcsF senses defects in LPS or other envelope components and initiates signaling through the RcsCDB phosphorelay (41). We overexpressed previously characterized, hyperactive alleles of *rcsF* (*rcsF_S17D_*) and *rcsC* (*rcsC_P484L_*) (42, 43) and monitored PF resistance by efficiency of plating assays. Although induction of either allele resulted in mucoid colony morphology consistent with elevated capsule production, it did not impact growth of WT or LPL-recycling mutants in the presence of PF (Fig. 3C). In addition, loss of the OM sensor RcsF, the response regulator RcsB, or the auxiliary protein RcsA failed to affect PF resistance of *lplT* and *aas* mutants (Fig. 3D). These results demonstrate that the Rcs response itself does not influence growth during LpxC inhibition. Instead, its activation in WT indicates extensive OM stress that is absent in ∆*lplT* and ∆*aas*; therefore, blocking LPL recycling protects the envelope from the stress normally induced by decreased LPS synthesis.

### PF resistance and reduction in envelope stress requires PldA

**Fig 4:**
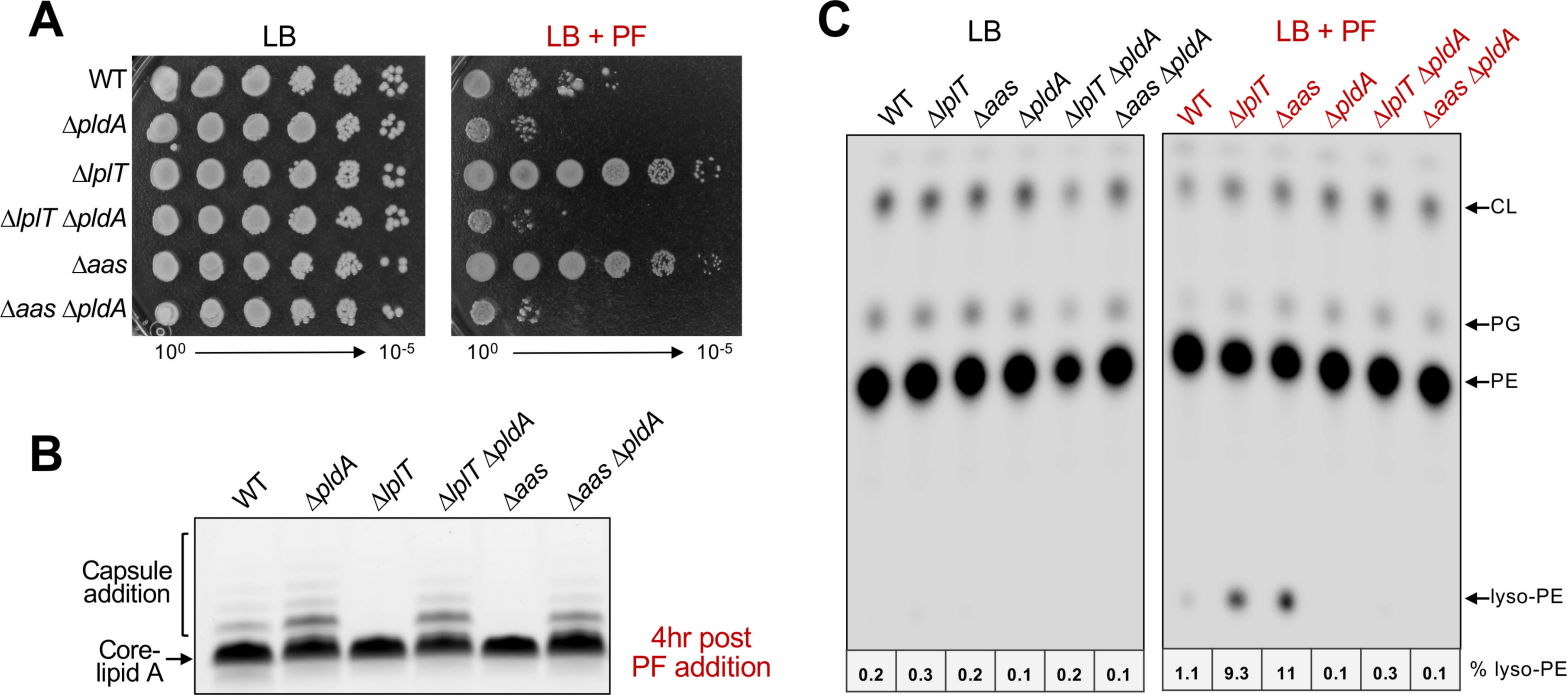
PF resistance and reduced envelope stress in LPL-recycling mutants are PldA-dependent. (**A**) Efficiency of plating assays comparing WT and LPL-recycling mutants lacking PldA in the absence or presence of 25 ng/mL PF. (**B**) LPS profiles of WT and ∆*pldA* strains grown with PF. Cultures were grown to an OD_600_ of 0.4 and exposed to 40 ng/mL PF for 4 h, and changes in LPS were assessed by SDS-PAGE. Capsule addition is indicated by a bracket. (**C**) TLC of extracted GPL species from WT or ∆*pldA* backgrounds with (red) and without (black) PF treatment. Following radiolabeling, GPLs were isolated and separated in the solvent chloroform:methanol:acetic acid (65:25:10, vol/vol/vol). Lipids (20,000 cpm per lane) were visualized by phosphorimaging, and densitometry within each lane was used to determine the percentage of lyso-PE present. Changes in lipid composition were similar for other biological replicates. Data are representative of a minimum of three biological replicates.

The reduced envelope stress observed in ∆*lplT* and ∆*aas* suggested that loss of LPL recycling triggers a protective response linked to changes in membrane lipid composition. Since PldA is a major source of LPL generation and its loss results in PF sensitivity of WT (Fig. 2B and C), we hypothesized that PldA activity could generate the lipid signal underlying PF resistance when LPL recycling is hindered. Deletion of *pldA* eliminated PF resistance of both *lplT* and *aas* mutants, reducing survival to the same level observed in a *pldA* single mutant (Fig. 4A). Moreover, LPS profiles of the double mutants revealed a restoration of capsule-modified LPS during PF treatment (Fig. 4B), demonstrating that suppression of the Rcs response in LPL-recycling mutants is also PldA-dependent.

To directly quantify LPL accumulation, bacteria were labeled with ^32^P_i_ to evaluate changes in lipid species. After growth, GPLs were isolated by Bligh-Dyer extraction, separated by thin-layer chromatography (TLC), and analyzed by phosphorimaging analysis as previously described (44). No differences in GPL profiles were observed for strains grown in LB. However, in the presence of PF, lyso-PE accumulated to ~1% of total GPLs in WT but increased by ~10-fold in LPL-recycling mutants (Fig. 4C). PE is the most abundant GPL in *E. coli,* serves as the acyl donor for lipoprotein maturation by Lnt, and is the primary substrate for PldA in the OM; thus, lyso-PE is the predominant LPL species detected. Previously, treatment of *E. coli* with CHIR-090 resulted in increased lyso-PE and lyso-PG levels (45). Additionally, CHIR-090 and PF have been shown to induce lyso-PE accumulation that is PldA-dependent (46). Likewise, we found that deletion of *pldA* nearly abolished lyso-PE accumulation (<0.5%), confirming that PldA is the dominant source of LPL generation during LpxC inhibition. Together, these data demonstrate that both PF resistance and the absence of envelope stress (i.e., Rcs activation) in LPL-recycling mutants depend on PldA activity. PldA-generated LPLs accumulate when recycling is blocked, linking phospholipase activity to a protective feedback response that restores OM homeostasis.

### Improved OM asymmetry of LPL-recycling mutants during inhibition of LPS synthesis

**Fig 5:**
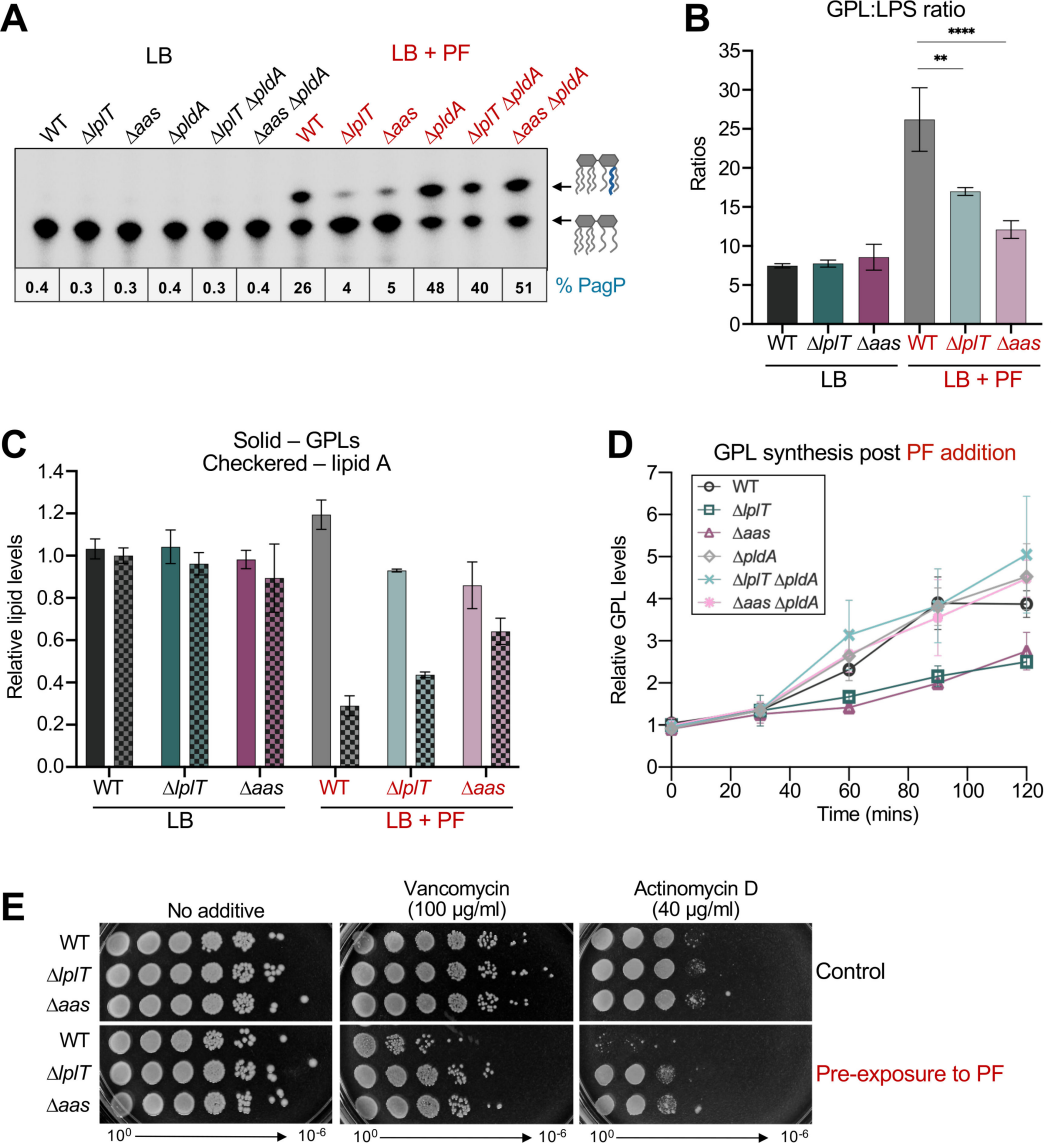
Loss of LPL recycling improves OM asymmetry during LpxC inhibition. (**A**) TLC of lipid A species from WT and ∆*pldA* backgrounds grown with (red) and without (black) PF. Each sample (5,000 cpms) was separated in the solvent chloroform:pyridine:88% formic acid:water (50:50:16:5, vol/vol/vol/vol). Lipids were visualized by phosphorimaging, and densitometry was used to calculate the percentage of hepta-acylated lipid A (i.e., PagP activity). Changes in lipid composition were similar for other biological replicates. (**B**) GPL-to-LPS ratios in the absence and presence of PF. Cultures were grown to an OD_600_ of 0.4 and exposed to 40 ng/mL PF for 2 h. GPLs and lipid A were extracted and quantified by scintillation counting. (**C**) Quantification of GPL and lipid A production following PF treatment. The cpms for each lipid sample were normalized to the total amount of ^32^P incorporation into the cell. GPL and lipid A levels relative to replicate 1 of WT are shown. Data for GPL-to-LPS ratios is from five biological replicates. (**D**) GPL synthesis assay during LpxC inhibition. Cells were grown with ^32^P_i_ to an OD_600_ of 0.4, when 40 ng/mL PF was spiked into cultures. At 30-min intervals over a 2 h period, aliquots were taken for GPL extraction and for the determination of total cellular ^32^P incorporation. Counts from extracted GPLs were first normalized to total radiolabel incorporation and then compared to WT GPL levels at 0 min and plotted over time. Data are representative of three biological replicates. (**E**) Efficiency of plating assays assessing changes in OM permeability. Cultures were grown to an OD_600_ of 0.4, treated with 30 ng/mL PF for 2 h, washed, and normalized by OD_600_. Serial dilutions were spotted on the indicated LB agar plates, and growth was evaluated. Error bars represent SD for bar graphs (**P-value ≤ 0.01; ****P-value < 0.0001). Data are representative of a minimum of three biological replicates.

Since LPL-recycling mutants lack an Rcs response when challenged with PF, we tested whether ∆*lplT* and ∆*aas* maintain OM asymmetry when LpxC is inhibited. The activity of the OM enzyme PagP provides a sensitive readout of changes in OM asymmetry. When the OM is disrupted, PagP transfers a palmitoyl group (C16:0) from a mislocalized GPL to LPS, generating a hepta-acylated lipid A anchor (24, 47) (Fig. 1). To monitor PagP activity, the cells were labeled with ^32^P_i_, and lipid A profiles were analyzed by TLC as previously described (48, 49). All strains show similar lipid A patterns in LB alone, while PF treatment resulted in the production of hepta-acylated lipid A in WT. ∆*lplT* and ∆*aas* cells showed markedly reduced PagP activity (4-5% vs. 26% in WT), indicating improved OM asymmetry under conditions when LPS synthesis is limited. This phenotype required PldA, as the same reduction in PagP activity was no longer observed when PldA was absent (Fig. 5A).

Because PF inhibits LPS synthesis in all strains, we considered whether improved asymmetry in ∆*lplT* and ∆*aas* reflected a corresponding decrease in GPLs. To test this, we quantified the steady-state GPL-to-lipid A ratio after growth with ^32^P_i_ for 2 h (Fig. 5B), allowing us to assess downstream effects on lipid synthesis following the initial reduction in LPS from PF exposure (Fig. 2E). The amount of total isotope incorporation was determined by subjecting an aliquot of the cell pellet of each strain to scintillation counting prior to lipid isolation. GPL and lipid A species were extracted separately, quantified, and the final yield of each lipid was normalized to total cellular ^32^P incorporation (see Fig. S4 for details). The final GPL-to-lipid A ratios provided a measure of the GPL-to-LPS balance, since the amount of lipid A directly reflects total LPS.

In the absence of PF, WT *E. coli* showed a GPL-to-LPS ratio of ~8, similar to *lplT* and *aas* mutants. PF treatment increased this ratio in all strains, but to varying degrees. As expected, a drastic increase was observed for WT (~26), while the GPL-to-LPS ratios of ∆*lplT* (~17) or ∆*aas* (~12) were substantially lower (Fig. 5B). Consistent with these values, total ^32^P-GPLs increased in WT during PF treatment, while this increase did not occur in recycling mutants; GPL levels were comparable between strains during growth in LB alone (Fig. 5C). Additionally, total ^32^P-lipid A increased in Δ*lplT* and Δ*aas* during PF exposure, while no difference was observed under non-stressed conditions. These steady-state measurements likely reflect a change in acyl-ACP utilization during LpxC inhibition. In WT, PF elicits an increase in GPL synthesis that consumes acyl-ACP intermediates. In contrast, the failure of LPL-recycling mutants to elevate GPL levels likely reduces acyl-ACP demand, potentially increasing substrate availability for lipid A production despite partial inhibition of LpxC.

To determine whether these steady-state differences reflect altered GPL synthesis dynamics, we quantified ^32^P-GPL production at 30-min intervals over a 2-h period after PF addition. As described above, total isotope incorporation was determined by scintillation counting, and GPL counts were normalized accordingly to account for any minor variance in growth. In WT, PF treatment caused a spike in GPL synthesis between 60 and 90 min. This transient increase in GPL production was absent in LPL-recycling mutants and was restored upon the loss of PldA (Fig. 5D). Differences were most evident at 90 min post-PF addition, where recycling mutants maintained significantly lower GPL levels compared to WT or ∆*pldA* strains (Fig. S5). Along with steady-state measurements (Fig. 5B and C), these data indicate that LPL-recycling mutants fail to elevate GPL synthesis when LPS is inhibited, resulting in a rebalancing of the GPL-to-LPS ratio.

Since ∆*lplT* and ∆*aas* have improved OM asymmetry in comparison to WT during LpxC inhibition, these mutants should display increased resistance to large antibiotics that do not normally penetrate the OM. Strains were treated with PF for 2 h and washed before serial dilutions were spotted on plates supplemented with vancomycin and actinomycin D. Since these antibiotics cannot enter cells via porins due to their large size (>1 kDa), increased sensitivity serves as a proxy for increased OM permeability. LPL-recycling mutants displayed increased growth in the presence of both antibiotics in comparison to WT (Fig. 5E), suggesting an improvement in OM asymmetry of ∆*lplT* and ∆*aas* compared to WT when LPS abundance is decreased.

### Disruption of LPL recycling rescues *yejM569* membrane defects

**Fig 6:**
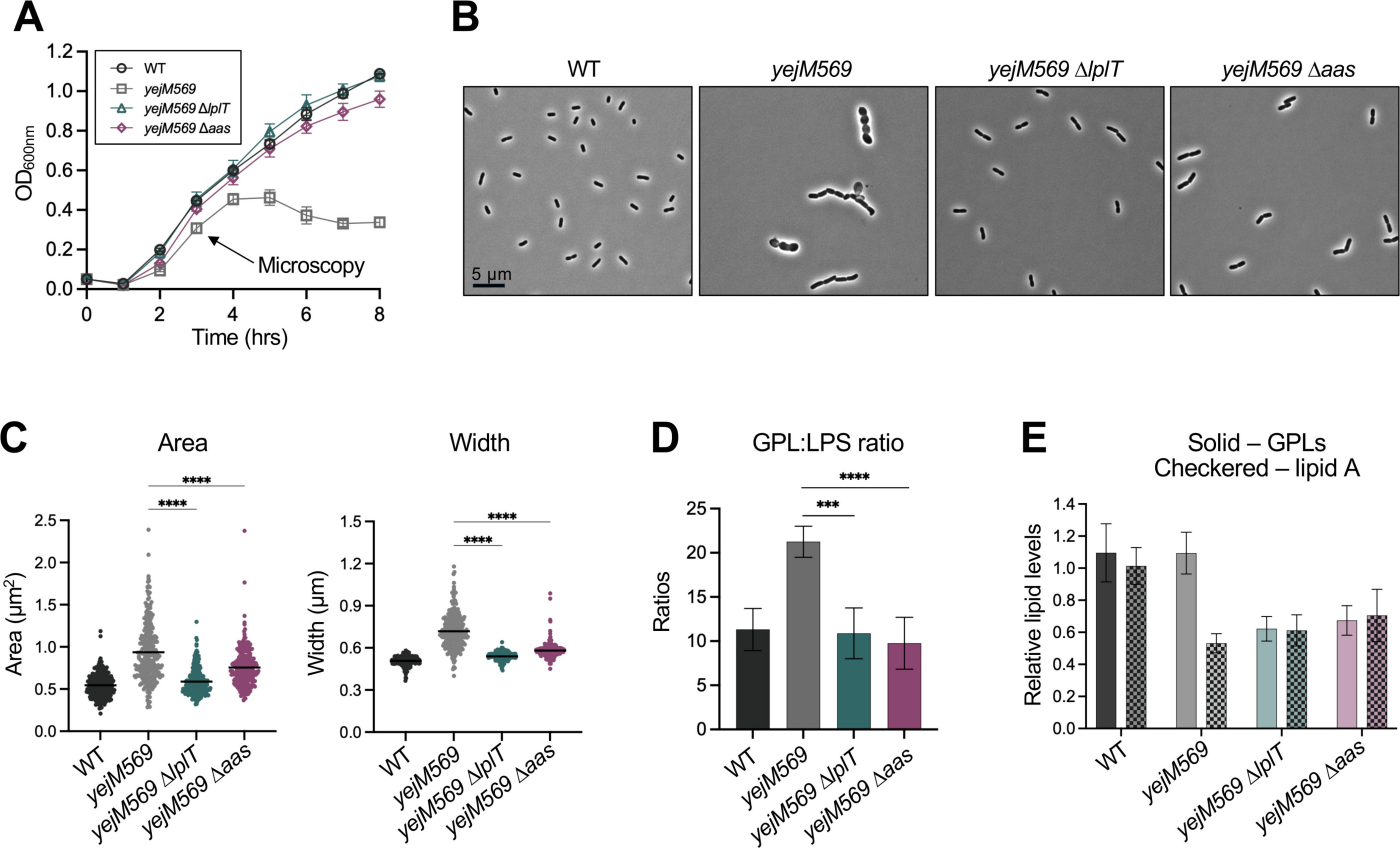
Deletion of *lplT* or *aas* rescues *yejM569* membrane defects. (**A**) Growth curve of WT and *yejM569* strains in LB at 42°C, with a starting OD_600_ of 0.005. Error bars represent SD and are not visible if smaller than the graphical symbol. (**B**) Phase-contrast microscopy after 3 h of growth at 42°C. Representative fields are shown, and additional fields of view are found in Fig. S6. (**C**) Cell area and width were measured for 274 cells using MicrobeJ software. Significance was determined using a Games-Howell test (****P-value < 0.0001). Microscopy was performed with biological duplicates. (**D**) GPL-to-LPS ratios of cultures grown for 4 h at 42°C. GPLs and lipid A were extracted from each culture and quantified by scintillation counting, and GPL-to-LPS ratios were determined. (**E**) GPL and lipid A counts were normalized to total cellular ^32^P incorporation. GPL and lipid A levels relative to replicate 1 of WT are shown with SD (***P-value ≤ 0.001, ****P-value ≤ 0.0001). Data for GPL-to-LPS ratios are obtained from five biological replicates.

To test whether the rescue of OM asymmetry observed in LPL-recycling mutants is specific to LpxC inhibition, we deleted *lplT* and *aas* in the *yejM569* genetic background (11–15). The *yejM569* allele encodes a YejM protein lacking its periplasmic domain, resulting in decreased LPS synthesis and increased antibiotic susceptibility (11, 50, 51). At 42°C, *yejM569* exhibits impaired growth, while the deletion of *lplT* or *aas* rescues growth to WT levels (Fig. 6A). Microscopy was performed 3 h after inoculation, when the growth defect of *yejM569* is first visible. At this stage, *yejM569* cells display pleomorphic morphology characterized by cell chaining, loss of rod shape, and increased cell size. In contrast, *yejM569* ∆*lplT* and *yejM569* ∆*aas* cells displayed only minor cell chaining and maintained morphology closer to that of WT (Fig. 6B; Fig. S6). Quantitative analysis confirmed that *yejM569* cells are significantly wider and larger than WT, and deletion of *lplT* or *aas* partially restored cell size (Fig. 6C).

We next asked if the rescue of *yejM569* growth and morphology is due to a decrease in GPL abundance that rebalances OM lipid composition. To test this, we assessed GPL-to-LPS ratios at 42°C. As previously reported, *yejM569* displayed a significantly higher ratio than WT (50) (Fig. 6D) and resulted directly from a decrease in LPS levels (Fig. 6E). Here again, deletion of *lplT* or *aas* rescued the GPL-to-LPS ratio driven primarily by a reduction in total GPLs (Fig. 6E). This decrease in GPL levels also restored OM asymmetry, as *yejM569* ∆*lplT* and *yejM569* ∆*aas* displayed enhanced growth in the presence of the large antibiotics vancomycin and actinomycin D at 42°C (Fig. S7). Overall, these data suggest disruption of LPL recycling improves fitness when LPS synthesis is limited and that this rescue is due to a cellular decrease in GPL levels.

We attempted to show dependence of *yejM569* rescue on PldA activity, which drives LPL accumulation, but found that *yejM569* ∆*pldA* mutants frequently acquire suppressor mutations in *lpxC* and *lapB* at temperatures above 30°C (Fig. S8). Two of the *lpxC* mutations identified here (*lpxC_R230L_* and *lpxC_V37G_*) were shown to increase LPS production or LpxC levels (11, 52), and disruption of LapB would be expected to hinder FtsH-mediated proteolysis of LpxC. These suppressors further support the importance of PldA function in the maintenance of OM asymmetry via GPL degradation and the generation of lipid signals.

### Modulating GPL synthesis alters PF resistance in LPL-recycling mutants

To strengthen the evidence that decreased GPL synthesis underlies PF resistance of ∆*lplT* and ∆*aas*, we manipulated fatty-acid flux to alter GPL levels within the cell. We first overexpressed the fatty-acid dehydratase FabZ, which elongates acyl-ACPs that preferentially enter GPL synthesis. FabZ and LpxA (Fig. 2A) share the substrate *R*-3-hydroxymyristoyl-ACP, placing these enzymes at a key branch point between GPL and LPS synthesis (53, 54). Overexpression of FabZ increases fatty-acid flux, generating more long-chain acyl-ACP donors (C16 and C18) for GPL synthesis and fewer medium-chain acyl-ACP donors (C12 and C14) for LPS synthesis (10, 55, 56).

**Fig 7:**
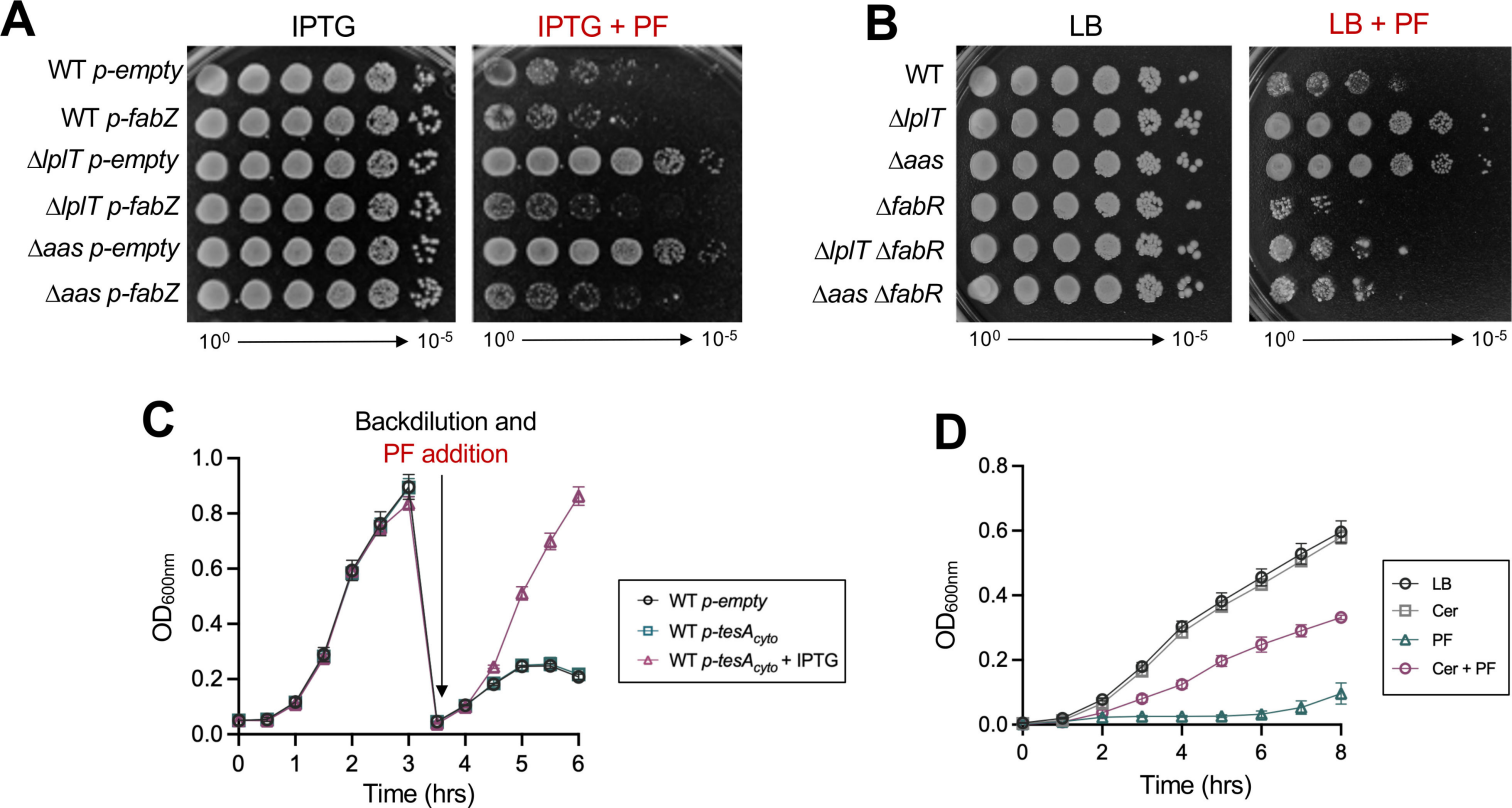
Modulating GPL synthesis alters PF resistance of ∆*lplT* and ∆*aas* mutants. (**A**) Efficiency of plating assays showing FabZ overexpression sensitizes LPL-recycling mutants to PF. Plates containing 50 µM IPTG and 25 ng/mL PF are indicated. (**B**) Efficiency of plating assays demonstrating that deletion of *fabR* reduces PF resistance. (**C**) Expression of cytoplasmic TesA (TesA_cyto_) enhances PF resistance of WT. Cultures were grown to an OD_600_ of ~0.8 in LB ± 50 µM IPTG and then back-diluted to an OD_600_ of 0.05 in LB + 20 ng/mL PF; 50 µg/mL ampicillin was used for plasmid maintenance. (**D**) Growth curve from a starting OD_600_ of 0.005 of WT supplemented with 1 µg/mL cerulenin (Cer) and/or 20 ng/mL PF as indicated. Growth was monitored in 96-well plates with orbital shaking, and error bars that represent SD are not visible if smaller than the graphical symbol. Data are representative of a minimum of three biological replicates.

*E. coli* suppressor mutants resistant to the LpxC inhibitor CHIR-090 harbor mutations that decrease FabZ activity; thus, overexpression of *fabZ* should decrease fitness during PF treatment (20). Indeed, increased FabZ expression reduced the growth of ∆*lplT* and ∆*aas* drastically during inhibition of LpxC (Fig. 7A). We next deleted *fabR*, which encodes a fatty acid biosynthesis regulator that represses expression of *fabA* and *fabB* for unsaturated fatty acid synthesis. Loss of FabR leads to increased long-chain unsaturated fatty acids and increased substrate for GPL synthesis (57). In both Δ*lplT* and Δ*aas*, deletion of *fabR* also reduced PF resistance (Fig. 7B). Overall, these data show that increasing production of long-chain fatty acids that feed into GPL synthesis negates PF resistance in LPL-recycling mutants.

Using multiple approaches, we next asked whether specifically lowering GPL synthesis in WT cells could reproduce the resistance phenotype of LPL-recycling mutants. The periplasmic protein TesA displays thioesterase and lysophospholipase activity (58–60). Expression of a cytoplasmic variant of TesA (TesA_cyto_) results in the cleavage of acyl-ACP and acyl-CoA molecules, which reduces the pool of substrate available for lipid synthesis (61, 62). Since TesA_cyto_ preferentially cleaves long-chain acyl-ACPs that feed into GPL synthesis (63), overexpression of TesA_cyto_ should impact fatty-acid flux and GPL synthesis to a greater extent than LPS synthesis (44). We induced the expression of *tesA_cyto_* in WT for 3 h and then back-diluted cultures into fresh LB supplemented with PF and assayed growth for 3 additional h. WT expressing TesA_cyto_ displayed elevated growth in comparison to cells without induction or the empty vector control (Fig. 7C). This result suggests that diminishing the pool of precursors for GPL synthesis increases growth in the presence of PF. We also used the antibiotic cerulenin to chemically inhibit fatty acid biosynthesis enzymes FabB and FabF to slow fatty-acid flux and downstream GPL synthesis (64, 65). Treatment with cerulenin causes a drastic reduction in long-chain fatty acids (C16) that feed into GPL synthesis and a mild increase in saturated, medium-chain fatty acids (C12, C14) that are used for lipid A synthesis (66). Thus, inhibition of fatty-acid flux by cerulenin impacts GPL synthesis to a greater extent than LPS synthesis. While WT fails to grow during LpxC inhibition, addition of just 1 µg/mL of the antibiotic partially rescues growth compared to PF treatment alone (Fig. 7D). Altogether, our data suggest that wild-type *E. coli* acquires PF resistance when substrate for GPL synthesis is depleted, which mimics the decrease in GPL levels that results in PF resistance of ∆*lplT* and ∆*aas*.

## DISCUSSION

The recent surge in multi-drug resistance in gram-negative bacteria underscores the urgent need for new antimicrobial targets and strategies to enhance drug penetration. Since LPS is required for growth in most gram-negative bacteria and for optimal OM barrier function, enzymes of the LPS synthetic pathway are attractive targets for antibiotic development (2). The LpxC inhibitor CHIR-090 and its various derivatives bind tightly to the active site of LpxC and halt growth at nanomolar concentrations (67). Although issues with toxicity and solubility have hindered their clinical use, LpxC inhibitors have proven valuable for dissecting cell envelope assembly and homeostasis (17, 19, 20). Here, using the LpxC inhibitor PF, we identify a lipid-based feedback mechanism that restores OM homeostasis when the recycling of LPLs is disrupted.

Together, the transporter LplT and the acyltransferase Aas recycle LPLs to reform GPLs within the IM. Deletion of *lplT* or *aas* conferred PF resistance (Fig. 2), and these phenotypes were complemented by WT alleles, but not by inactive variants of LplT or Aas (Fig. S1D). PF treatment induced robust PagP activity in WT that was mitigated by the loss of LplT or Aas (Fig. 5A). Because PagP only uses mislocalized GPLs at the cell surface as substrate, the reduction in PagP activity indicated improved OM asymmetry, which was confirmed by the lower GPL-to-LPS ratios of recycling mutants during PF treatment (Fig. 5B). This restoration of asymmetry improved OM integrity, as shown by decreased activation of the Rcs envelope stress response (Fig. 3) and increased resistance to large antibiotics (Fig. 5E). Although cellular LPS levels decreased in LPL-recycling mutants during PF treatment, a concomitant decrease in GPL abundance (Fig. 5C and D) balanced lipid composition and enhanced OM barrier function.

Importantly, this rescue of OM asymmetry was not limited to chemical inhibition of LpxC. Deletion of *lplT* or *aas* also restored growth of the *yejM569* strain that produces less LPS due to impaired stabilization of LpxC (11). In this background, loss of LPL recycling largely restored cell morphology defects associated with loss of YejM regulatory function and rescued the GPL-to-LPS ratio by lowering GPL production (Fig. 6). Consistent with a causal role for reduced GPL synthesis in protection against PF, directly increasing GPL flux by overexpressing *fabZ* or deleting *fabR* sensitized LPL-recycling mutants to PF. Furthermore, reducing fatty acid synthesis and GPL flux in WT via genetic or chemical means enhanced growth during LpxC inhibition (Fig. 7). Together, these findings suggest that resistance of LPL-recycling mutants arises from a regulated decrease in GPL synthesis, which rebalances OM composition.

PF resistance, the lack of envelope stress (Rcs activation), and reduced PagP activity in LPL-recycling mutants were all dependent on PldA, suggesting that PldA and its enzymatic products are essential for this adaptive response (Fig. 4 and 5). During PF treatment, PldA served as the dominant source of lyso-PE that accumulates and was required to prevent a spike in GPL synthesis in ∆*lplT* and ∆*aas* (Fig. 4C and 5D). However, whether LPLs or free fatty acids generated by PldA serve as the signal to reduce GPL synthesis is a central question.

A role for PldA-derived fatty acids in envelope homeostasis was previously defined in mutants defective in the Mla retrograde GPL transport system, which normally removes mislocalized GPLs from the outer leaflet of the OM to maintain lipid asymmetry (68). When this pathway is disrupted, GPLs accumulate at the cell surface and activate PldA (69). In that context, May and Silhavy showed that PldA-generated fatty acids are converted by FadD into acyl-CoAs, which stabilize LpxC and drive LPS production (68). In this scenario, LpxC is catalytically intact, and FadD is essential for the response. In contrast, our Tn-seq data indicated that disruption of *fadD* improves fitness in the presence of PF (~40-fold increase, FDR *P*-value 1.11E−07) (Data Set S1), and direct genetic analysis confirmed that deletion of *fadD* in ∆*lplT* or ∆*aas* backgrounds does not diminish PF resistance (data not shown). These findings indicate that the acyl-CoA-dependent signaling route identified by May and Silhavy is inactive when LpxC is chemically inhibited. Because PF directly blocks LpxC catalysis, stabilizing the enzyme is likely no longer advantageous, and fatty-acid-derived signaling provides no benefit. Instead, LPLs generated by PldA accumulate and appear to function as feedback signals that suppress GPL synthesis and restore the GPL-to-LPS balance. Thus, PldA serves a dual role, as a degradative enzyme that clears mislocalized GPLs from the OM and as a generator of two distinct lipid messengers that adjust membrane biogenesis according to the metabolic state of LpxC.

While LPLs have well-established signaling roles in eukaryotes, analogous functions have not been defined in bacteria (25). Nevertheless, LPL accumulation is observed across diverse bacterial species during exposure to environmental stressors. In *E. coli* and *Yersinia pseudotuberculosis*, heat shock induces an increase in cellular lyso-PE, which is proposed to act as a molecular chaperone that prevents protein aggregation and induces protein renaturation (70, 71). In *Campylobacter jejuni*, a dramatic rise in lyso-PE is proposed to promote growth and motility in low oxygen conditions (72). Finally, lyso-PE accumulates to a staggering 25%–30% of GPLs in *Vibrio cholerae* when grown in the presence of bile (73). These examples highlight that LPL accumulation is a common bacterial response to membrane stress and may alter cell envelope properties or signal adaptive remodeling. To identify if disruption of LPL recycling is a conserved resistance mechanism to LpxC inhibition, we assessed the growth of *Acinetobacter baumannii* strain 5075 and Tn insertion mutants of *lplT* and *aas* (74) in the presence of PF. Growth was monitored in 96-well plates due to the increased resistance of *A. baumannii* to LpxC inhibition (19), and the prohibitive cost of PF. *lplT* and *aas* Tn mutants displayed elevated growth compared to wild-type 5075 during PF treatment, while only a minor growth advantage was apparent in LB (Fig. S9). These data indicate a conserved role for LPL accumulation in membrane homeostasis when LPS is limiting.

Our findings suggest a role for LPLs in coordinating GPL synthesis with OM homeostasis. The receptor or effector through which the LPL signal exerts this regulatory influence remains unknown, and it is possible that regulation does not require a dedicated sensor. Instead, accumulation of LPLs within the IM may alter local membrane properties—such as curvature stress, bilayer packing, or lateral pressure—which, in turn, modulate fatty acid flux and GPL synthesis (75). Determining whether LPLs directly influence GPL biosynthetic enzymes or act indirectly through changes in membrane physical state will be an important focus of future work. Notably, because suppression of GPL synthesis occurs in both *aas* and *lplT* mutants, efficient translocation of LPLs to the cytoplasmic leaflet does not appear to be strictly required for this regulatory effect. Clarifying the localization and dynamics of LPL accumulation will therefore be essential for resolving the mechanism of this regulatory response.

Our data support a model in which PldA functions as a central hub that generates lipid signals to maintain OM homeostasis. When GPLs are mislocalized to the cell surface, PldA degrades them, producing both free fatty acids and LPLs. We propose that the cell interprets these products differently, depending on the status of LpxC and the capacity of the LplT-Aas recycling system. When LpxC is active, and LPL recycling keeps pace, free fatty acids are converted by FadD into acyl-CoA, which stabilizes LpxC and promotes LPS synthesis. In contrast, when LpxC activity is chemically inhibited or directly suppressed by LapB (YciM) binding, or when LPL recycling becomes saturated, LPLs accumulate and act as feedback signals that slow GPL synthesis. Thus, the same initial membrane stress event, GPL exposure at the surface, can elicit opposite outcomes depending on the metabolic state of the cell. We hypothesize that the acyl-CoA route enhances LPS production when LpxC is active, whereas the LPL route restrains GPL synthesis when LpxC activity or recycling capacity is limited. Under steady-state growth, both signals likely operate at low levels to maintain envelope balance. Small amounts of free fatty acids and LPLs are continuously produced during lipoprotein maturation and membrane remodeling, providing ongoing feedback that fine-tunes lipid metabolism and preserves OM asymmetry.

Future work should focus on identifying the molecular targets that respond to LPL accumulation and determining how these lipid cues are sensed and transduced. In parallel, clarifying how the LPL and acyl-CoA signals intersect with the YejM-LapB-FtsH network, which modulates both LpxC stability and catalytic activity, will help define the hierarchy of lipid signaling in envelope homeostasis. These insights may reveal new vulnerabilities for disrupting OM stability in antibiotic-resistant pathogens.

## MATERIALS AND METHODS

### Strains and growth conditions

Strains, plasmids, and primers used in this study are listed in Data Set S3 in the supplemental material. LB broth and agar plates were created using BD Difco Luria-Bertani (LB) broth or agar. Where appropriate, growth media were supplemented with ampicillin (50 or 100 µg/mL), kanamycin (30 µg/mL), D-glucose (0.2%), PF-5081090 (as indicated), isopropyl-β-D-1-thiogalactopyranoside (IPTG; 50 or 100 µM), or with other supplements as indicated in figure legends. Unless stated otherwise, all cultures were grown at 37°C. Growth curves were conducted in 5 mL LB in glass tubes. Every hour, 200 µL of the culture was transferred to a polystyrene 96-well plate (Corning), and the OD_600_ was measured using a BioTek Epoch 2 plate reader. Growth curves in 96-well plates were completed in 200 µL LB in the plate reader with orbital shaking (365 rpm), with OD_600_ measurements taken every 30 mins.

### Strain and plasmid construction

Deletion strains were generated using the Keio collection and generalized P1 transduction into W3110 *E. coli*, as previously described (35). When necessary, kanamycin resistance cassettes flanked with FLP recognition target sites were removed as previously described by transformation with pCP20, which encodes the FLP recombinase (76). The ∆*aas-lplT* strain was generated via recombineering using *E. coli* strain DY330 and the aas-lplT-pKD4 primers, and the deletion allele was transduced into W3110 (77). Plasmids were created using restriction cloning. All inserts were amplified using PFU Turbo polymerase AD (Agilent). BamHI and EcoRI restriction enzymes, Antarctic phosphatase, and T4 ligase (NEB) were used for digestions, plasmid dephosphorylation, and ligations, respectively. pWSKI was used for all cloning (78). pWSKI-fabZ was synthesized by GenScript. Site-directed mutagenesis was conducted as previously described to construct hyperactive *rcs* alleles (rcsF S17D and rcsC P484L primers) and inactive *lplT* (lplT D30A primers) and *aas* (aas H36A primers) alleles (79). Whole plasmid sequencing was performed by Plasmidsaurus using Oxford Nanopore Technology with custom analysis and annotation.

### Transposon sequencing (Tn-seq)

A saturated Tn library of *E. coli* K-12 strain W3110 was generated as previously described (49). Briefly, a donor strain carrying the mariner Tn was mated with W3110, and ~400,000 exconjugants were collected and stored at −80°C. The library was grown in triplicate in 50 mL LB ± 20 ng/mL PF. gDNA was extracted from cell pellets and sheared, and poly-G tails were added for library builds. Amplicon libraries were prepared for the Illumina HiSeq platform and sequenced at Azenta (2 × 150 bp, ~350M paired-end reads). Sequencing analysis was done using Qiagen CLC Genomics Workbench.

### Efficiency of plating assays

Overnight cultures were normalized to an OD_600_ of 1.0 and serially diluted in a 96-well plate. Dilutions were spotted onto plates using a sterile replicator (Sigma Aldrich), and the plates were incubated at 37°C for approximately 18 h, unless stated otherwise.

### RNA sequencing

Triplicate overnight cultures were back-diluted to an OD_600_ 0.05 and incubated at 37°C to an OD_600_ ~0.2, when 30 ng/mL PF was spiked into the indicated cultures. Cultures were grown to a final OD_600_ of 0.5, and 1 mL of culture was mixed with 4 mL of RNAlater (Invitrogen). Samples were vortexed, incubated at room temperature for 5 min, and pelleted at 6,000 × *g* for 10 min. Library preparation, rRNA depletion, and Illumina RNA sequencing were performed at SeqCenter. Analysis was performed using Qiagen CLC Genomics Workbench.

### LPS gels

Overnight cultures were back-diluted to an OD_600_ of 0.05 and incubated at 37°C. At OD_600_ ~0.4, 40 ng/mL PF was spiked into the indicated cultures, and 1 mL of culture was harvested at 1 or 4 h after PF addition. Cell pellets were resuspended in 250 µL of PBS with 1% SDS and boiled for 15 min. Samples were centrifuged at 17,000 × *g* for 5 min, and the supernatant was transferred to a new tube. Protein concentration of each sample was measured using the Pierce BCA assay. Samples were normalized by protein concentration in 1× LDS buffer + 4% BME and incubated with 0.3 mg/mL of proteinase K (NEB) at 55°C overnight. Samples were boiled for 15 min and vortexed vigorously before loading what was the equivalent of 6 µg of total protein (prior to proteinase K treatment) onto a 4%–12% Bis-Tris gel (Invitrogen). Gels were run at 150 V for 1 h, and instructions of the Pro-Q Emerald LPS gel stain kit (Molecular Probes) were followed. Gels were visualized using the Bio-Rad ChemiDoc MP Imaging System.

### Immunoblots

Overnight cultures were back-diluted to an OD_600_ of 0.05 and incubated at 37°C. At an OD_600_ of ~0.4, 40 ng/mL PF was spiked into indicated cultures; 1.5 mL of culture was harvested after 0 or 2 h of outgrowth. Cell pellets were prepared as described in “LPS gels” excluding proteinase K treatment, and 3 µg of total protein was loaded onto a 10% Bis-Tris gel (Invitrogen) and the Amersham ECL Plex fluorescent molecular weight standards (Cytiva) was used. Gels were run at 150 V for 1 h and transferred to 0.2 µm PVDF membranes at 10 V for 1 h using the Trans-Blot SD semi-dry transfer system (Bio-Rad). Membranes were blocked in 2% milk-PBST (PBS + 0.2% Tween-20) for 1 h. LpxC was visualized using the rabbit polyclonal anti-LpxC antibody (1:5,000) (80) paired with the Cyanine5 goat anti-rabbit secondary antibody (1:10,000) (Invitrogen). The RNA polymerase (RNAP) β mouse antibody (1:10,000) (Biolegend), paired with the Cyanine5 goat anti-mouse secondary antibody (1:10,000) (Invitrogen), was used for the detection of the RNAP loading control. Blots were visualized using the Bio-Rad ChemiDoc MP Imaging System. Densitometry of LpxC bands was normalized to that of the RNAP loading control.

### LpxC stability assays

Overnight cultures were back-diluted to an OD_600_ of 0.05 in 30 mL LB and incubated at 37°C. At an OD_600_ of ~0.4, 40 ng/mL PF was spiked into cultures. After a 2 h outgrowth, chloramphenicol (200 µg/mL) was added to cultures to halt protein synthesis. At the indicated time points following antibiotic addition, OD_600_ was measured, and 1.5 mL of culture was immediately pelleted at 10,000 × *g* for 2 min and frozen at −80°C. Pellets were prepared for western blot as described in “Immunoblots” with 10 µg protein loaded per gel.

### Lipid extractions

Strains were back-diluted to an OD_600_ of 0.05 in LB + 2.5 µCi/mL of ^32^P_i_ and grown to an OD_600_ of ~0.4, when 40 ng/mL PF was added to indicated cultures. After an outgrowth of 2 h, the cells were pelleted and washed with PBS. Pellets were resuspended in 1:2:0.8 chloroform:methanol:water (vol/vol/vol) (i.e., single-phase Bligh-Dyer mixture) and incubated at room temperature for 20 min. After centrifugation, the supernatant was transferred to a new tube for the extraction of GPLs, while the pellet was processed for the extraction of lipid A, as previously described (48, 49). GPL and lipid A counts were determined by scintillation using a PerkinElmer liquid scintillation analyzer. Lipids were spotted on 20 × 20 TLC silica gel 60 plates and developed in chloroform:methanol:acetic acid (65:25:10, vol/vol/vol) solvent mixture to separate GPL species and a chloroform:pyridine:88% formic acid:water (50:50:16:5, vol/vol/vol/vol) mixture to separate lipid A species. Plates were exposed to a phosphorimaging screen overnight and imaged using the Amersham Typhoon Biomolecular Imager (Cytiva). Densitometry of lipid species was performed using ImageQuant software.

### GPL-to-LPS ratios

WT, ∆*lplT*, and ∆*aas* were grown as previously described in “Lipid extractions.” WT and *yejM569* strains were grown in LB at 42°C for 4 h. Washed cell pellets were resuspended in 5 mL of PBS, and 100 µL was counted by scintillation to determine total ^32^P incorporation into the cells (total counts) for each sample. After the extraction of GPLs and lipid A species, lipids were resuspended in 500 µL of chloroform:methanol (4:1, vol/vol); 200 µL was counted to determine the total GPLs and lipid A in each sample. The GPL and lipid A counts were normalized to total counts for each processed sample, allowing for comparisons across strains. Division of GPL by lipid A counts yielded GPL-to-LPS (lipid A) ratios. An unpaired *t*-test was used to determine statistical significance between the strains, and data were acquired for five biological replicates.

### GPL synthesis assay

Strains were back-diluted to an OD_600_ of 0.05 in 7 mL LB + 5 µCi/mL of ^32^P_i_ and grown to an OD_600_ of ~0.4, when 40 ng/mL PF was added to the indicated cultures. Every 30 min post-PF addition, 100 µL of culture was pelleted, washed, and counted by scintillation to determine total ^32^P incorporation. For GPL extraction, 500 µL of the same culture was added directly to a mixture containing 2 mL chloroform, 2 mL methanol, and 1.3 mL of PBS to generate a two-phase Bligh Dyer mixture. After extraction, GPLs were resuspended in 500 µL of chloroform:methanol (4:1, vol/vol) and counted by scintillation to determine total GPLs in each sample. The GPL counts were normalized to total ^32^P counts for each processed sample, allowing for comparisons across strains over time. An unpaired *t*-test was used to determine statistical significance between strains.

### Phase-contrast microscopy

Strains were back-diluted to an OD_600_ of 0.005 and grown at 42°C. At 3 h, 2 µL of culture was spotted on a glass slide and covered with a 3% agarose pad. Cells were imaged with the Nikon Eclipse TI2-E microscope using the 100× objective lens with immersion oil. Cell size analysis was completed using MicrobeJ, and a Brown-Forsythe and Welch ANOVA test assuming standard deviations were not equal. Statistical significance was determined using a Games-Howell test.

### Suppressor analysis

*yejM569* ∆*pldA* and *yejM569* ∆*aas* ∆*pldA* strains were generated and maintained at 30°C. Overnight cultures were grown at 37°C for 24 h. Cells were pelleted and sent for library preparation and whole genome sequencing using SeqCenter.

## Data Availability

Additional replicate data can be found in the Zenodo repository (10.5281/zenodo.18839946).

## SUPPLEMENTAL MATERIAL

Data Set 1 mbio.00567-26-s0001.xlsx PF Tn-seq data.

Data Set 2 mbio.00567-26-s0002.xlsx RNA seq data.

Data Set 3 mbio.00567-26-s0003.xlsx Strains, plasmids, and primers.

Supplemental Material mbio.00567-26-s0004.pdf Fig. S1 to S9 and captions for Data Sets S1 to S3.

ASM does not own the copyrights to Supplemental Material that may be linked to, or accessed through, an article. The authors have granted ASM a non-exclusive, world-wide license to publish the Supplemental Material files. Please contact the corresponding author directly for reuse.
