# Supplementary material for: Lysophospholipid signaling coordinates outer membrane homeostasis in Escherichia coli
Source: mBio. 2026 Apr 14;17(5):e00567-26. doi: 10.1128/mbio.00567-26 (PMC13170283; doi:10.1128/mbio.00567-26)
Supplement: Supplemental Material — Fig. S1 to S9 and captions for Data Sets S1 to S3. [file mbio.00567-26-s0004.pdf]

Supporting information for manuscript "Lysophospholipid signaling coordinates outer membrane homeostasis in *Escherichia coli.*"

Authors: Gerald R. Enverso[a] and M. Stephen Trent[a, b,*]

[a]Department of Microbiology, College of Arts and Sciences and [b]Department of Infectious Diseases, College of Veterinary Medicine; University of Georgia, Athens, Georgia, USA

SUPPLEMENTAL FIGURES AND LEGENDS

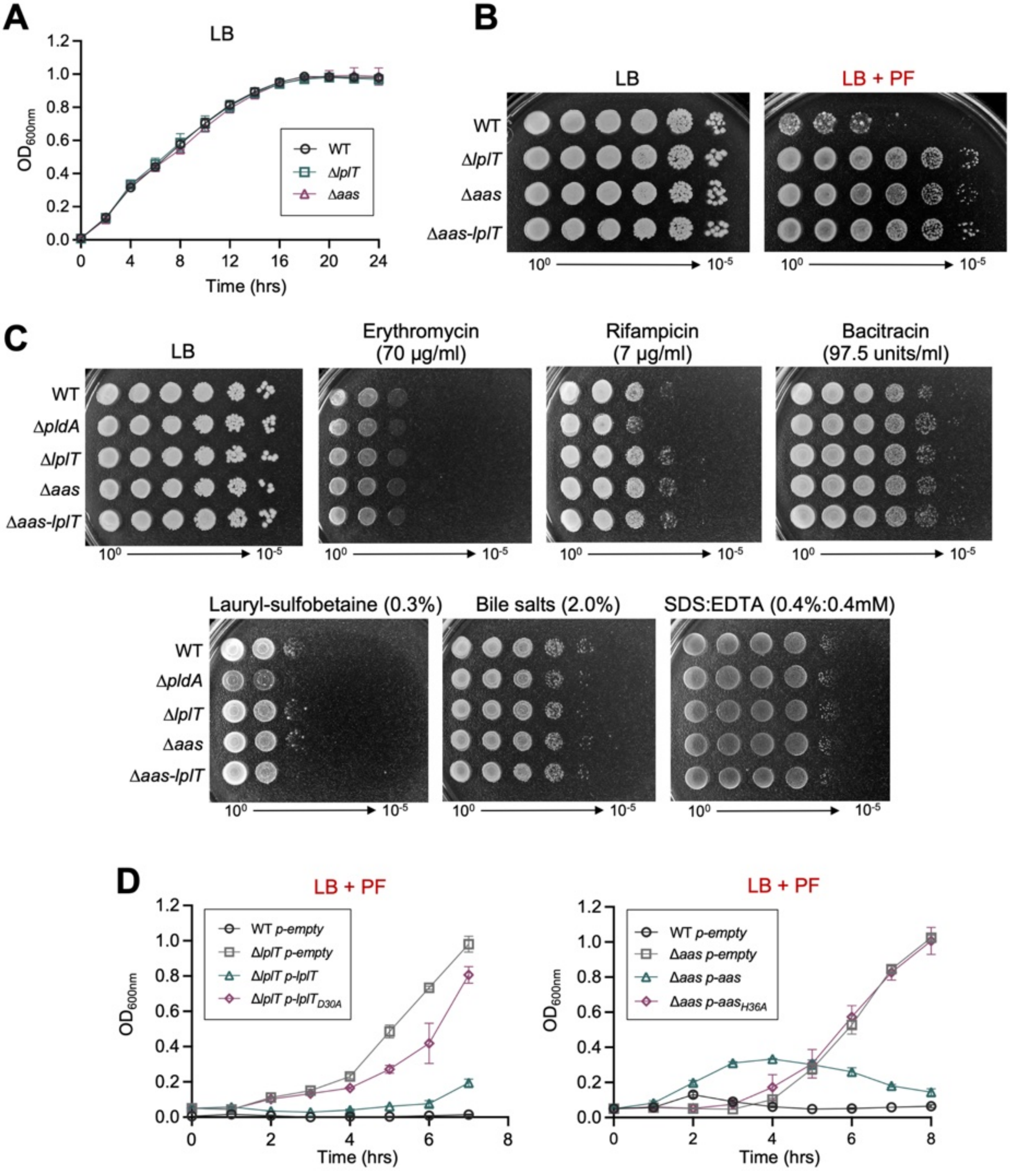

FIG S1 Characterization of LPL-recycling mutants. **(A)** Growth of WT and LPL-recycling mutants in LB from starting OD_{600nm} 0.005. Growth was monitored in 96-well plates with orbital shaking. **(B)** Efficiency of plating assay to assess PF resistance of Δ*aas-lplT* mutant in comparison to single mutants. Serial dilutions were plated on LB and LB + 25 ng/mL PF plates. **(C)** Efficiency of plating assay of LPL-recycling mutants in the presence of various antibiotics and detergents. **(D)** Growth curves of WT and LPL-recycling mutants with 20 ng/mL PF. Cultures were back-diluted to starting OD_{600nm} 0.05 in LB and 100 µg/mL ampicillin was included for plasmid maintenance. Complementation of Δ*lplT* required only basal expression, whereas Δ*aas* required induction with 100 µM IPTG. Expression of inactive variants of LplT or Aas failed to complement. All data are representative of a minimum of three biological replicates. For growth curves, error bars represent SD and are not visible when smaller than the graphical symbol.

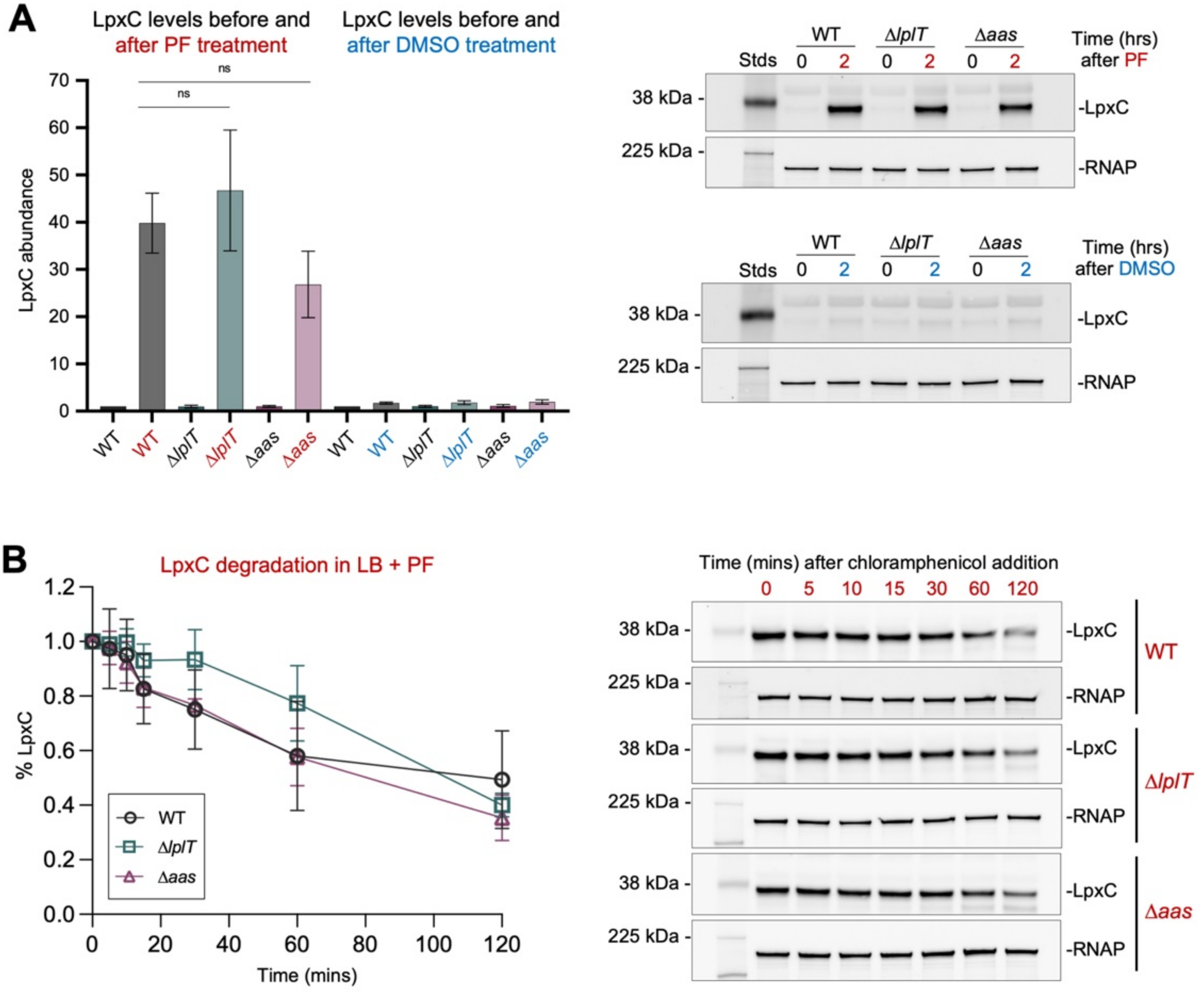

FIG S2 LpxC levels and stability are comparable in WT and LPL-recycling mutants during PF exposure. **(A)** PF treatment increases LpxC abundance. Cultures were grown to OD_{600nm} 0.4, when 40 ng/mL PF or DMSO (vehicle control) was added. Cells were pelleted after 0 or 2 hrs of growth and prepared for immunoblot to detect LpxC or RNAP. Densitometry of LpxC was normalized to RNAP (loading control) and displayed LpxC levels are relative to WT at 0 hrs. **(B)** LpxC is stabilized by PF treatment. Cultures were treated with 40 ng/mL PF for 2 hrs, then protein synthesis was stopped by addition of 200 μg/mL chloramphenicol. At the indicated time points, cells were pelleted and prepared for immunoblot to detect LpxC or RNAP. Densitometry of LpxC was normalized to RNAP and % LpxC is relative to LpxC levels at 0 mins. Representative blots for both panels are shown with data representative of a minimum of three biological replicates.

Fold changes of select Rcs-regulated genes during PF treatment

Gene	WT FC	Δ*lplT* FC
flhC	-9.94	-1.85
flhD	-18.94	-2.56
wcaF	+376.59	+1.76
wcaE	+736.23	+1.70
wza	+214.83	+1.67
gmd	+106.69	+1.57
yjbE	+471.73	+4.10
yjbG	+97.94	-1.23
cpsB	+37.85	+2.64
cpsG	+32.65	-1.08
osmB	+376.46	+2.28
osmY	+60.38	+3.94
rcsA	+10.56	-1.23

FIG S3 Expression of Rcs-regulated genes during LpxC inhibition. Weighted fold changes (FC) of select, Rcs-regulated genes from triplicate RNA-Seq data of WT and Δ*lplT* during PF treatment. FDR-corrected *p*-values are less than 0.05.

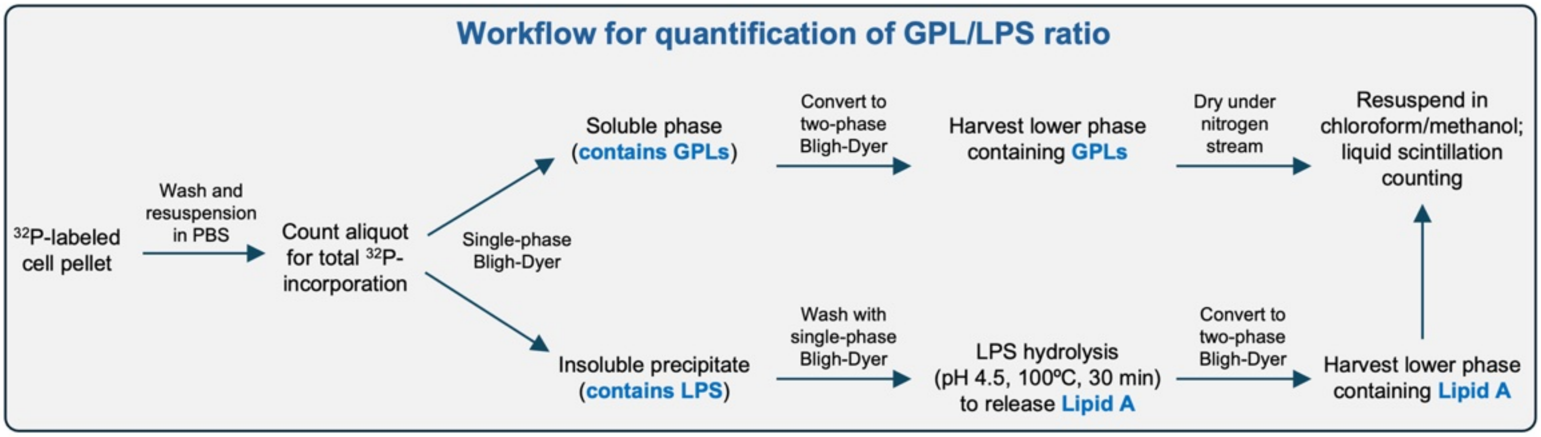

FIG S4 GPL-to-LPS ratios workflow. Cells were grown in the presence of 2.5 µCi/mL of $^{32}P_i$, harvested by centrifugation, and the final cell pellets resuspended in 5 mL of PBS. To determine total ^{32}P incorporation (total counts) for each sample, 100 µL of each suspension was subjected to scintillation counting. The remaining cells were pelleted and resuspended in 5 mL of single-phase Bligh-Dyer mixture and incubated for 20 mins. Under these conditions, GPLs remain soluble while LPS precipitates because of its large carbohydrate domain. After centrifugation, the soluble phase containing GPLs was transferred to a new tube and converted to a two-phase Bligh-Dyer mixture; GPLs were collected from the lower phase. For lipid A isolation, the insoluble pellet was washed with single-phase Bligh-Dyer mixture, resuspended in 50 mM sodium-acetate (pH 4.5) containing 1% SDS, and boiled at 100°C for 30 mins to release lipid A from LPS. After cooling, the solution was converted to a two-phase Bligh-Dyer mixture, and lipid A was extracted from the lower phase. Final lower phases from both GPL and lipid A extractions were dried under nitrogen, resuspended in 500 µL of chloroform:methanol (4:1, v/v), and 200 µL was counted by scintillation to determine GPL and lipid A cpms. GPL-to-LPS ratios were calculated by dividing GPL cpms by lipid A cpms. Additional information is provided in the “GPL-to-LPS ratios” section of Materials and Methods.

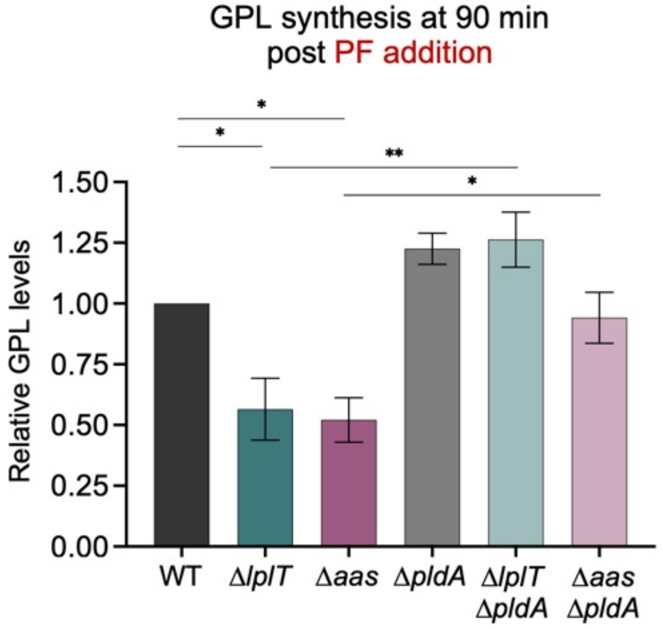

FIG S5 GPL synthesis assay at 90-min timepoint. GPL levels at 90 mins post-PF treatment. The cpms for each sample were normalized to the total amount of ^{32}P-incorporation into the cell. GPL levels are relative to the WT strain. Error bars represent SD (**p*-value ≤ 0.05 ***p*-value ≤ 0.01). Data are representative of three biological replicates.

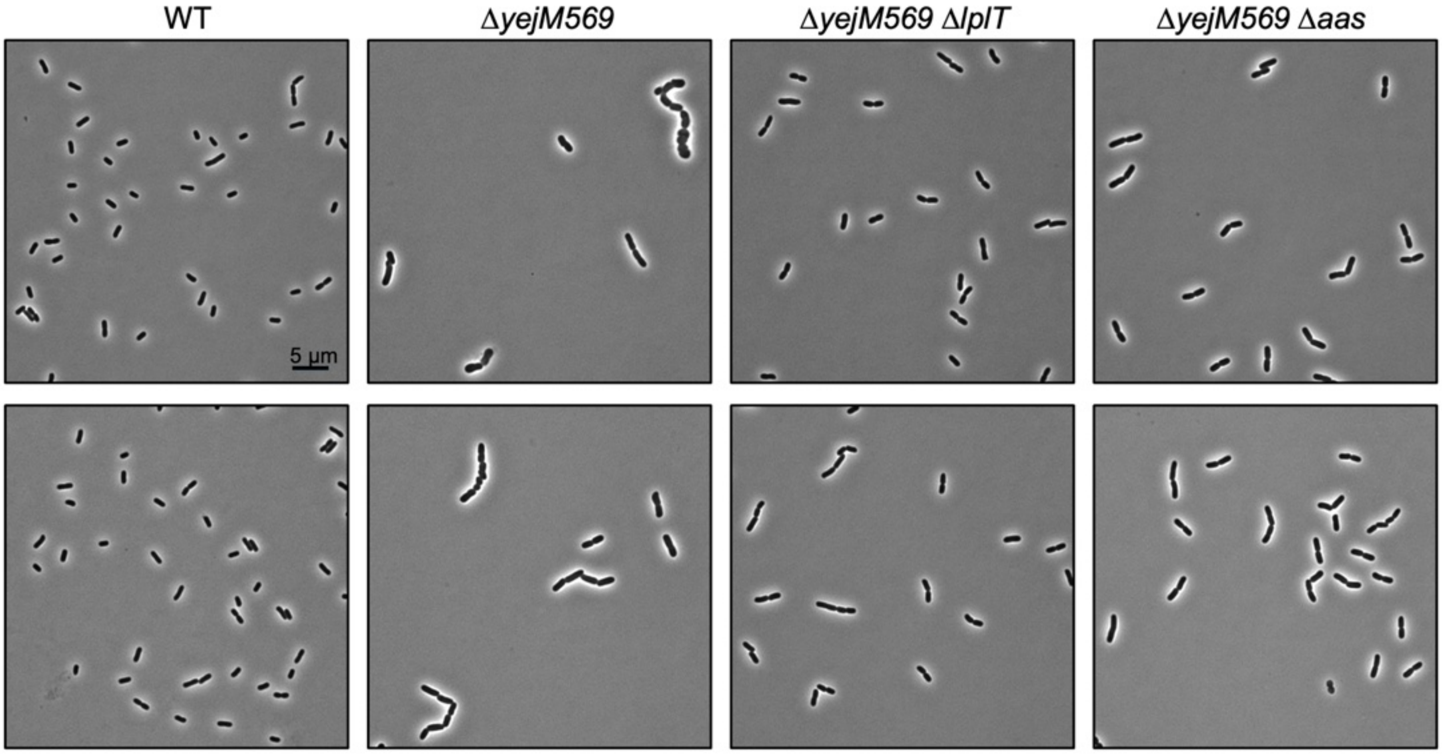

FIG S6 Additional fields of view of phase-contrast microscopy of *yejM569* and *yejM569* LPL-recycling mutants. As shown in **Fig 6A**, microscopy was performed after growth at 42°C for 3 hrs.

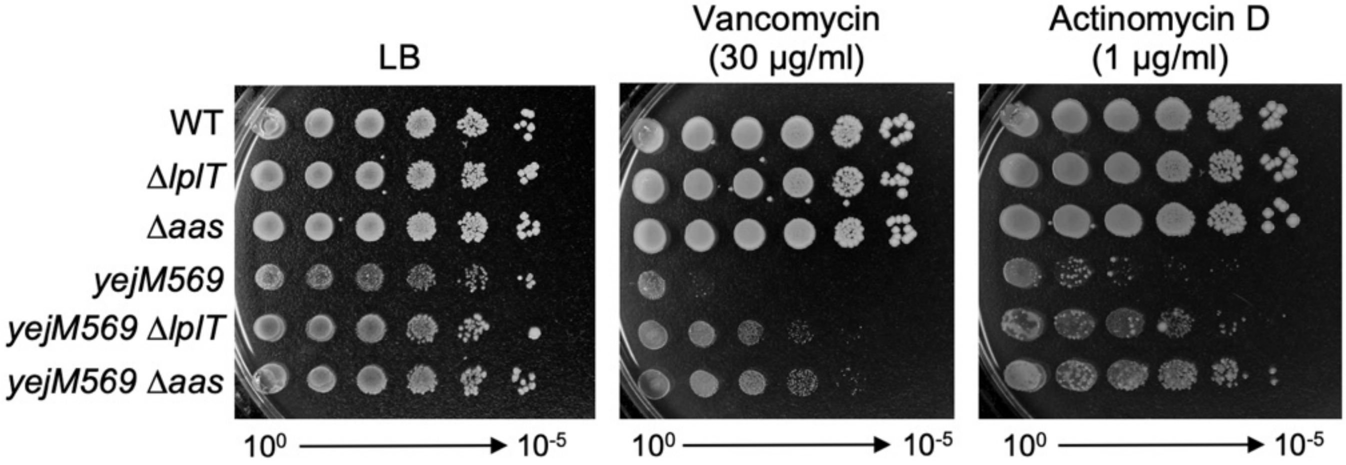

FIG S7 Efficiency of plating assays of *yejM569* strains at 42°C. WT and *yejM569* strains were plated on LB in the absence and presence of large antibiotics to test rescue of OM permeability from deletion of *lplT* or *aas*. Data are representative of a minimum of three biological replicates.

Parent strain	Suppressor number	Suppressor description
yejM569 Δ*pldA*	1	*lapB* M315I
	2	*lpxC* R230L
	3	*lapB* 12 AA deletion in C-terminus
yejM569 Δ*aas* Δ*pldA*	1	*lpxC* V37G
	2	*lpxC* D43 codon change

FIG S8 *yejM569* Δ*pldA* suppressors. Suppressors were acquired from overnight cultures of *yejM569* Δ*pldA* strains grown at 37°C.

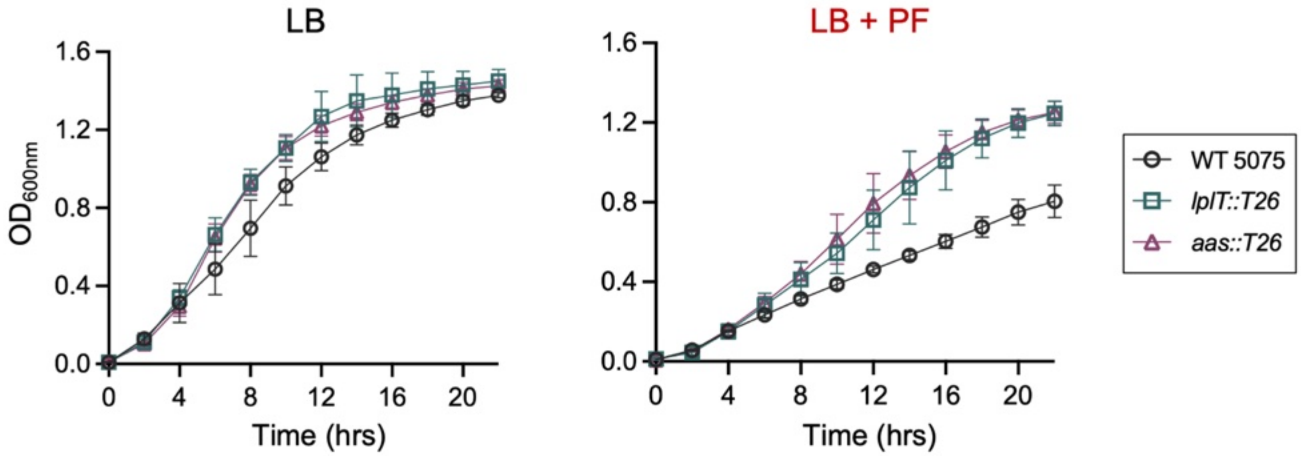

FIG S9 Disruption of LPL recycling benefits growth of *A. baumannii* during LpxC inhibition. Growth of *A. baumannii* wild-type strain 5075 and LPL-recycling Tn mutants in LB alone or LB containing 12.5 µg/mL PF. Cultures were grown at 37°C with starting OD_{600nm} 0.005. Growth was monitored in 96-well plates and error bars show SD and are not visible if smaller than graphical symbol. Data are representative of three biological replicates.

DATA SETS

Data Set S1 (separate file). Tn-seq analysis of wild-type *E. coli* K-12 (strain W3110) during challenge with PF. Fold-change comparisons and FDR *p*-values from triplicate Tn-seq data are shown.

Data Set S2 (separate file). RNA-seq analysis of both wild type and Δ*lplT* in the presence and absence of PF. Raw reads for each sample (triplicate data) are provided in Tabs 2, 4, 6, 8. Fold-change comparisons between samples and FDR *p*-values are provided in Tabs 1, 3, 5, and 7. Descriptions for individual data sets are indicated in Tab labels.

Data Set S3 (separate file). Strains, plasmids, and primers used in this manuscript. Tab 1 provides a list of strains used in this study. Strains assessed by whole-genome sequencing has been indicated. Plasmids and primers used in this study are listed in Tabs 2 and 3, respectively.

DATA AVAILABILITY

Additional replicate data can be found in the Zenodo repository (**10.5281/zenodo.18839946)**
